# cGAS/STING sensing in dendritic cells discriminates between daptomycin sensitive and resistant *Staphylococcus aureus* clinical isolates

**DOI:** 10.1016/j.isci.2026.115854

**Published:** 2026-04-22

**Authors:** Timothy Patton, Nazneen Jahan, Jhih-Hang Jiang, Xenia Kostoulias, Ee Shan Pang, Rachel J. Lundie, Katherine Balka, Rajan Venkatraman, Viola Oorschot, Joan Clark, Sharifeh Askary, Peck Tan, Angus Shoppee, Georg Ramm, Anton Y. Peleg, Dominic De Nardo, Meredith O’Keeffe

**Affiliations:** 1 Monash Biomedicine Discovery Institute, and Department of Biochemistry and Molecular Biology, Monash University, Clayton, VIC 3800, Australia; 2 Department of Microbiology and Immunology, The University of Melbourne at the Peter Doherty Institute for Infection and Immunity, Melbourne, VIC 3000, Australia; 3 Infection Program, Monash Biomedicine Discovery Institute, Department of Microbiology, Monash University, Clayton, VIC 3800, Australia; 4 Now at Commonwealth Serum Laboratories, Melbourne, VIC 3000, Australia; 5 Monash Ramaciotti Centre for Cryo Electron Microscopy, Monash University, Clayton, VIC 3800, Australia; 6 Now at Electron Microscopy Core Facility, European Molecular Biology Laboratory, Heidelberg, Germany; 7 Department of Infectious Diseases, Alfred Hospital and Central Clinical School, Monash University, Melbourne, VIC 3000, Australia

**Keywords:** Natural sciences, Biological sciences, Microbiology

## Abstract

Antimicrobial-resistant infections present significant threats to public health, predicted to cause 10 million fatalities annually by 2050. Of these infections, one of the most frequent is methicillin-resistant *Staphylococcus aureus* (MRSA). During therapeutic exposure to last-line antibiotics, including daptomycin, MRSA often acquires genetic mutations conferring resistance*.* Resistance is typically mediated through structural alterations to the bacterial cell wall and membrane, which we have previously shown, concomitantly impede the activation of responding dendritic cells (DCs). Here, we demonstrate how MRSA acquisition of daptomycin resistance impedes innate immune sensing by DC. We show that the acquisition of daptomycin resistance reduced phagocytosis by DC, and that it impaired the recognition of resistant isolates via the cytoplasmic cGAS/STING pathway, affecting the production of cyclic dinucleotides produced by both the host and the bacterium. Our research uncovers a mechanism by which antibiotic resistance mutations can simultaneously hinder the innate immune system’s ability to recognize bacteria.

## Highlights

• MRSA is recognized by dendritic cells through the cGAS/STING pathway
• Lack of STING signaling reduces the uptake of MRSA by dendritic cells
• MRSA that are daptomycin resistant produce less cyclic dinucleotides
• Daptomycin-resistant MRSA fail to effectively activate STING in dendritic cells

## Graphical abstract

**Figure:**
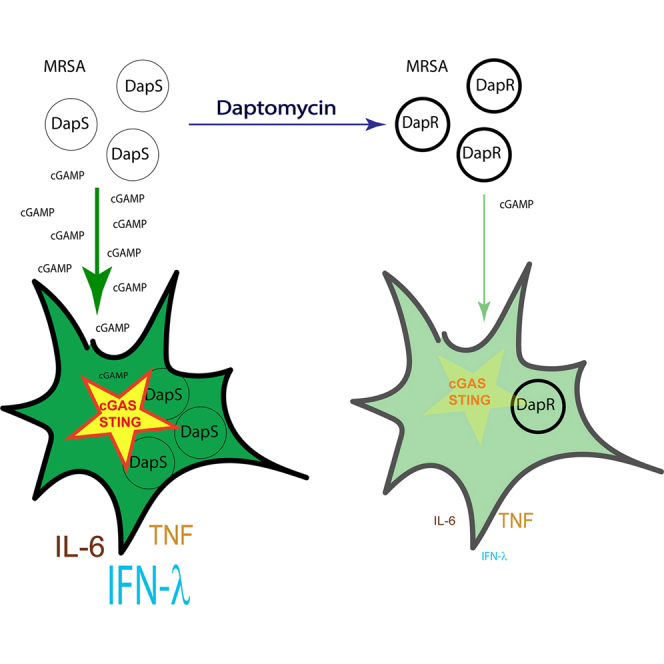

## Introduction

Antimicrobial resistant infections currently present one of the most significant threats to public health as we emerge from the COVID-19 pandemic. Globally, antimicrobial-resistant infections lead to more than 700,000 fatalities each year, and this number is predicted to rise to 10 million fatalities per year by 2050.^1^^,^^2^ Of all hospital acquired infections, *S. aureus* represents one of the most frequently diagnosed, comprising more than one-fifth of all blood-stream infections.^3^ These infections are typically described based on their susceptibility to the antimicrobial methicillin, which provides a useful delineation given infection with methicillin resistant *S. aureus* (MRSA) has a mortality rate almost double that of methicillin-sensitive *S. aureus* (MSSA).^4^ MRSA infection is generally treated with either of the last-line antibiotics daptomycin or vancomycin. However, MRSA often accumulates genetic mutations conferring resistance to these last-line antibiotics during therapeutic exposure^5^^,^^6^; which is associated with complex and more persistent infections and therapeutic failure.^5^^,^^6^^,^^7^^,^^8^^,^^9^ Considering the rising rates of antimicrobial resistance, particularly to last-line antibiotics, understanding the immune response to *S. aureus* is critical.

We have recently demonstrated a differential capacity of clinical MRSA isolates to stimulate the activation of mouse DC generated from bone marrow cells *in vitro* with Flt3-ligand.^10^ Using a series of paired daptomycin exposed isolates, we showed that the acquisition and accumulation of common daptomycin resistance mutations simultaneously impede recognition by murine DC.^10^ Further, these very same daptomycin resistance mutations also compromise neutrophil recruitment to a localised site of infection in a zebrafish model and in *in vitro* transwell assays.^11^ In both instances, we attribute the impaired innate sensing of the daptomycin-resistant (DapR) MRSA daughter strains to alterations in the structure and composition of the bacterial cell envelope (cell wall and membrane), which is driven by these daptomycin mutations.^5^^,^^11^ Understanding the precise mechanisms by which these DapR MRSA isolates modulate innate DC responses is of clear importance in order to build an understanding of the altered immune responses to multidrug-resistant MRSA infections.

The innate cell sensing of MRSA, a gram-positive bacterium, is thought to involve peptidoglycan recognition at the cell surface through TLR2. Upon phagocytosis, the bacterial DNA is potentially recognized via TLR9 and endosomal TLR3, 7/8, and TLR13, all of which potentially recognize RNA adducts from phagocytosed MRSA (reviewed in^12^). Indeed, TLR knockout mice infected with MRSA strains indicate a role for TLR2 in managing bacterial load.^13^ The importance of endosomal TLRs in MRSA mouse infections is less clear, with TLR9 deficiency shown both to improve,^14^ or make no change,^13^^,^^15^ in MRSA bacterial clearance and mortality. TLR7-deficient mice similarly have shown no change in infection control, while the combined deficiency of TLRs 7, 9, and 13 greatly enhanced bacterial loads in skin infection models.^13^

Phagocytosed MRSA has been shown to escape to the cytoplasm^16^ where DNA and/or cyclic dinucleotides could be recognized via the cyclic GMP-AMP synthase (cGAS)/stimulator of interferon response cGAMP interactor (STING) pathway.^12^ cGAS binds dsDNA in the cytoplasm, which activates cGAS to convert ATP and GTP into the metazoan-specific cyclic dinucleotides (CDNs) 2′3′-cGAMP for STING recognition and activation.^17^^,^^18^ STING is also able to bind CDNs from alternative sources, including CDNs produced and released by bacteria such as 3′3′-cGAMP.^19^ In both global^20^ and myeloid-specific^21^ STING-deficient mice, an MRSA-induced pneumonia infection model (USA300 strain) displayed increased bacterial load in the lungs, while, conversely, in skin infections, STING-deficient mice were able to more efficiently clear bacteria.^22^ However, whole animal STING knockout studies are open to interpretation, given the importance of STING in maintaining tonic IFN signaling through microbiota DNA sensing^23^ and potentially influencing innate immune responses.

To understand responses to MRSA clinical isolates, it is of importance to decipher recognition at the cellular level. Here, using clinical isolates of daptomycin-exposed MRSA (Table 1),^5^^,^^10^^,^^11^ we examine how changes at the bacterial surface impede innate immune sensing by murine conventional DC1 (cDC1). We show that there is significantly reduced uptake of DapR clinical isolates by DC in comparison to their daptomycin susceptible (DapS) parent isolates.

Moreover, we demonstrate that the impaired recognition of DapR isolates is regulated through the cGAS/STING pathway. We observed that STING activation is significantly higher in DC upon sensing DapS compared to DapR MRSA, commensurate with higher production of cytokines and type III IFNs (IFN-λs), which were ablated in response to DapR MRSA. Of note, IFN-λ production was entirely dependent on cGAS-STING. Importantly, compared to DapS strains, DapR isolates induce less cGAS activation measured as mammalian CDNs production from murine DC. Bacteria produce CDNs as a response to “danger” or stress responses in the form of nutrient limitation,^24^ phage infection,^25^ envelope stress,^26^ antibiotics,^27^ and/or temperature^28^ or variations in salt balance and pH.^29^ We found that DapR isolates produced substantially less bacterial CDN to directly activate STING. When exposed to media containing β-lactam antibiotics, DapS, but not DapR isolates, produced substantial levels of CDNs, suggesting that the altered cell envelope acquired during daptomycin therapy may shield the bacteria against antibiotic-induced stress responses.

Our work sheds light on ways in which antibiotic resistance mutations can alter stress sensing in bacteria and simultaneously impair mammalian innate immune recognition. We identify the activation of the mammalian cGAS/STING pathway by DapS MRSA, but not DapR MRSA, as the major difference between the sensing of these paired clinical isolates. These findings have important implications for the development of the next generation of antimicrobial therapies and the fight against the growing threat of AMR.

## Results

### Daptomycin-resistant MRSA are inefficiently internalized by DC

**Figure 1:**
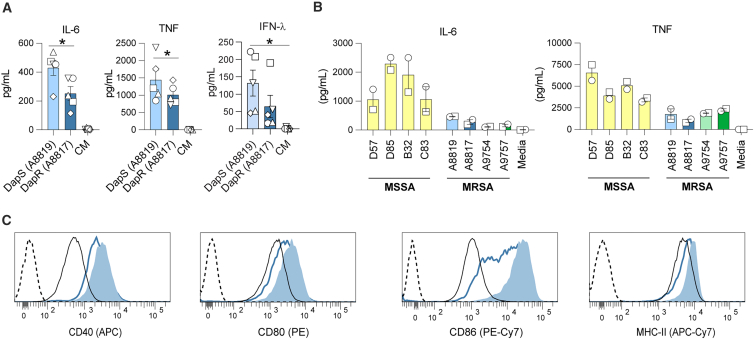
The MutuDC cell line models the primary cDC1 response to *S. aureus* (A) Cytokine secretion (pg/mL) by FACS sorted splenic cDC1 at 18 h post stimulus with DapS (A8819; light blue), DapR (A8817; dark blue), MRSA (MOI of 10), or media alone control (black). Results from 3 independent experiments are expressed as the mean ± SEM, with each symbol (circle, square, diamond, and triangle) representing paired experimental replicates (*n* = 5). Statistical significance determined using a paired *t* test and reported as: ∗p ≤ 0.05, ∗∗p ≤ 0.01, ∗∗∗p ≤ 0.001. (B) Cytokine secretion (pg/mL) by MutuDC stimulated with four independent clinical isolates of MSSA (D57, D85, B32, and C83; yellow) and two series of paired MRSA isolates, including the A8819/A8817 clinical pair (as in A) and a second pair corresponding to DapS A9754 (light green) and DapR A9757 (dark green). Results from 2 independent experiments are expressed as the mean ± range, with each symbol (circle and square) representing paired experimental replicates (*n* = 2). (C) Expression of activation markers by MutuDC stimulated with MRSA isolates, as in A, showing DapS (A8819; light blue shaded), DapR (A8817; dark blue trace), MRSA (MOI of 10), or media alone (black solid trace). Unstained control sample is shown for each marker (black dashed trace). Data shown from one experiment, representative of three independent experiments (*n* = 3).

We previously found that MRSA clinical isolates were sensed predominantly by the conventional DC subset 1 (cDC1), among DC generated *in vitro* with flt3-ligand (FLDC).^10^ Here we extended this finding, demonstrating predominant cytokine responses and cell surface activation by both mouse splenic cDC1 (Figures S1 and S2) and the splenic cDC1 cell line, MutuDC (Figure 1). Consistent with our published data on FLDC,^10^ primary and immortalized splenic cDC1 displayed a hierarchy of inflammatory cytokine responses to *S. aureus* clinical isolates, with the strongest responses to MSSA, followed by MRSA, and the weakest responses to daptomycin-resistant (DapR) MRSA (Figures 1A, 1B, and S2). IL-6, TNF, and IFN-λ were all significantly higher in response to DapS MRSA compared with DapR MRSA (Figure 1A). DapR MRSA also failed to induce the high levels of CD86 induced by DapS MRSA on the surface of MutuDC (Figure 1C). These data suggest that MSSA strains display a greater array of pathogen-associated molecular patterns (PAMPs) to activate the DC, and that this pool decreases, first with the ubiquitous resistance to methicillin, and then with the acquisition of daptomycin resistance.

**Figure 2:**
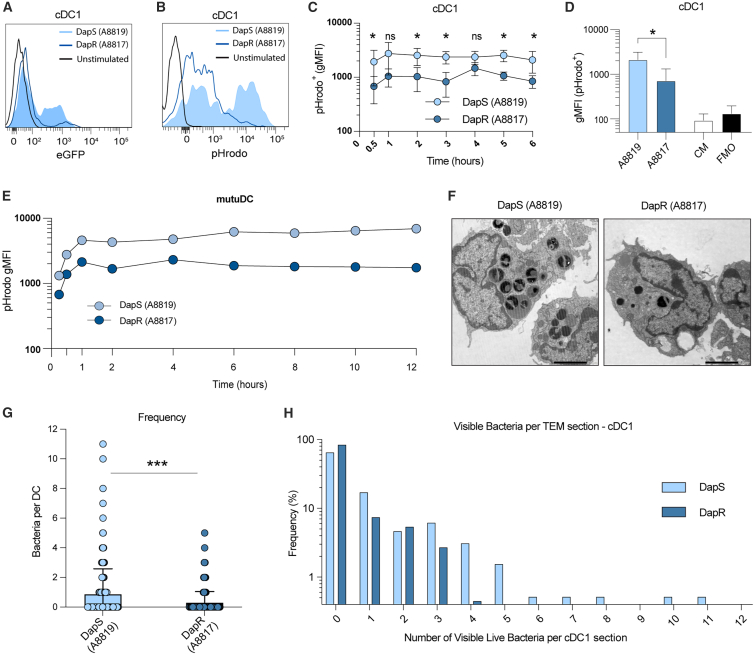
Quantitation of MRSA internalization by splenic DC reveals that DapR MRSA are taken up less efficiently than DapS MRSA (A and B) Representative histograms showing uptake of DapS A8819 MRSA (blue shaded), DapR MRSA (dark blue trace), or media alone (black trace) by cDC1 following 3 h incubation period. Relative MRSA internalization is measured through flow cytometric quantitation of eGFP (A) and pHrodo (B) as surrogate markers for uptake. (C) Relative pHrodo labeled MRSA internalization by splenic cDC1 over 6 h following stimulation with DapS A8819 (light blue) or DapR A8817 (dark blue). Showing the mean ± SEM of pHrodo geometric mean fluorescence intensity (gMFI) from four individual mice (*n* = 4) pooled from 3 independent experiments. Statistical significance determined at each time point using a ratio paired *t* test and reported as indicated by ∗*p* ≤ 0.05, ∗∗*p* ≤ 0.01, and ∗∗∗*p* ≤ 0.001. (D) pHrodo labeled MRSA internalization as in C, showing just the 3 h timepoint. Statistical significance determined using a ratio paired *t* test and reported as indicated by ∗*p* ≤ 0.05, ∗∗*p* ≤ 0.01, and ∗∗∗*p* ≤ 0.001. (E) Relative pHrodo labeled MRSA internalization by MutuDC over 12 h following stimulation with DapS A8819 (light blue) or DapR A8817 (dark blue). Showing the mean and range of gMFI from 2 independent experiments (*n* = 2). (F) Transmission electron micrograph of fixed and Epon-embedded DC sections corresponding to FACS-sorted cDC1 following 8-h MRSA stimuli with either DapS A8819 (left) or DapR (A8817) (right) at an MOI of 10. Images were acquired on a JEOL TEM electron microscope with between 200× and 30,000× magnification. Images represent DC with the highest number of visibly internalized MRSA for each isolate. Scale bars are representative of 2 μm. Data shown from 1 experiment representative of two independent experiments (*n* = 2). (G) cDC1 internalization of DapS (A8819) and DapR (A8817) MRSA, enumerated by eye in blinded DC sections prepared as in F. Each circle represents the number of visible MRSA enumerated per DC, in a complete dataset of sections comprising cDC1 stimulated with DapS A8819 MRSA (*n* = 194), and DapR A8817 MRSA (*n* = 435). Significance reflective of an unpaired two-tailed *t* test using Welch's correction, reported as indicated by ∗*p* ≤ 0.05, ∗∗*p* ≤ 0.01, and ∗∗∗*p* ≤ 0.001. (H) Frequency of DapS (A8819) and DapR (A8817) MRSA in cDC1 sections enumerated as in F. Data shown are representative of two independent experiments on FACS sorted cDC1 (4 and 8 h stimulation periods, respectively), with trends representative of an additional experiment on unsorted bulk splenic DC at 8 h (*n* = 3).

Given the difficulty in treating and clearing MRSA infections that become resistant to the last-line antibiotic Daptomycin, it is important to unravel the mechanisms hampering the recognition of DapR MRSA by DC. To this end, we examined the uptake of DapS and DapR paired clinical strains by *ex vivo* splenic DC. We utilized clinical MRSA isolates expressing eGFP^11^ and co-cultured these bacteria with *ex vivo* splenic DC using acquisition of the eGFP signal by DC as a surrogate marker for DC-MRSA association (Figure 2A, S3A, and S3B). We found that DapS was rigorously taken up by the cDC1 (Figure 2A) and also by spleen cDC2 and minimally by pDC (Figures S3A and S3B). However, DapR MRSA was, in comparison, poorly taken up by the DC (Figures 2A and S3B). To confirm that the acquisition of eGFP signal was representative of genuine bacterial internalization, we further labeled these isolates with the pH-sensitive dye pHrodo, which is only detectable at a low pH, such as within phagolysosomes (Figures 2B and 2C). Similar to our finding with GFP-positive isolates, we observed less uptake of pHrodo-labelled DapR MRSA strains (A8817 and A9764) than DapS MRSA (A8819 and A9763) in all spleen DC (Figures 2C, S3C, and S3D). Over a 12-h time course, we found that the acquisition of eGFP by DC correlated with that of pHrodo at early time points, probably due to the lack of eGFP fluorescence at low pH,^30^ GFP signal gradually declined from 4 h onward (Figure S3B), while pHrodo signal plateaued (Figures 2C and S3C). The MutuDC cDC1 cell line showed similar, differential uptake of pHrodo-labelled DapS and DapR MRSA (Figure 2E). Together, these data confirmed differential uptake of DapS and DapR MRSA into low pH compartments.

We next utilised conventional electron microscopy to validate the findings of the flow-based assay, comparing DapS A8819 and DapR A8817 internalization by the cDC1s (Figure 2F). Consistent with results obtained by flow cytometry Figures 2C, 2D, and S3D), we found that cDC1 internalized significantly more DapS A8819 than DapR A8817 MRSA per cell on average (Figure 2G), with a higher frequency of visible internalized DapS MRSA per section than for DapR MRSA (Figure 2H). Approximately 17% of the analyzed cell sections contained internalized DapR MRSA, about 35% contained DapS MRSA (Figure 2H). Thus, upon the acquisition of Daptomycin resistance, DapR *S. aureus* strains are phagocytosed less efficiently than their parental daptomycin-sensitive strain.

### TLR2 signaling contributes to DC sensing of MRSA but not uptake

We found that the uptake of DapR MRSA by DC is less than that of DapS MRSA. As bacterial uptake may be only one factor in the diminished response of DC to DapR MRSA, we next sought to understand which pattern recognition receptors are utilized in sensing the paired clinical isolates.

**Figure 3:**
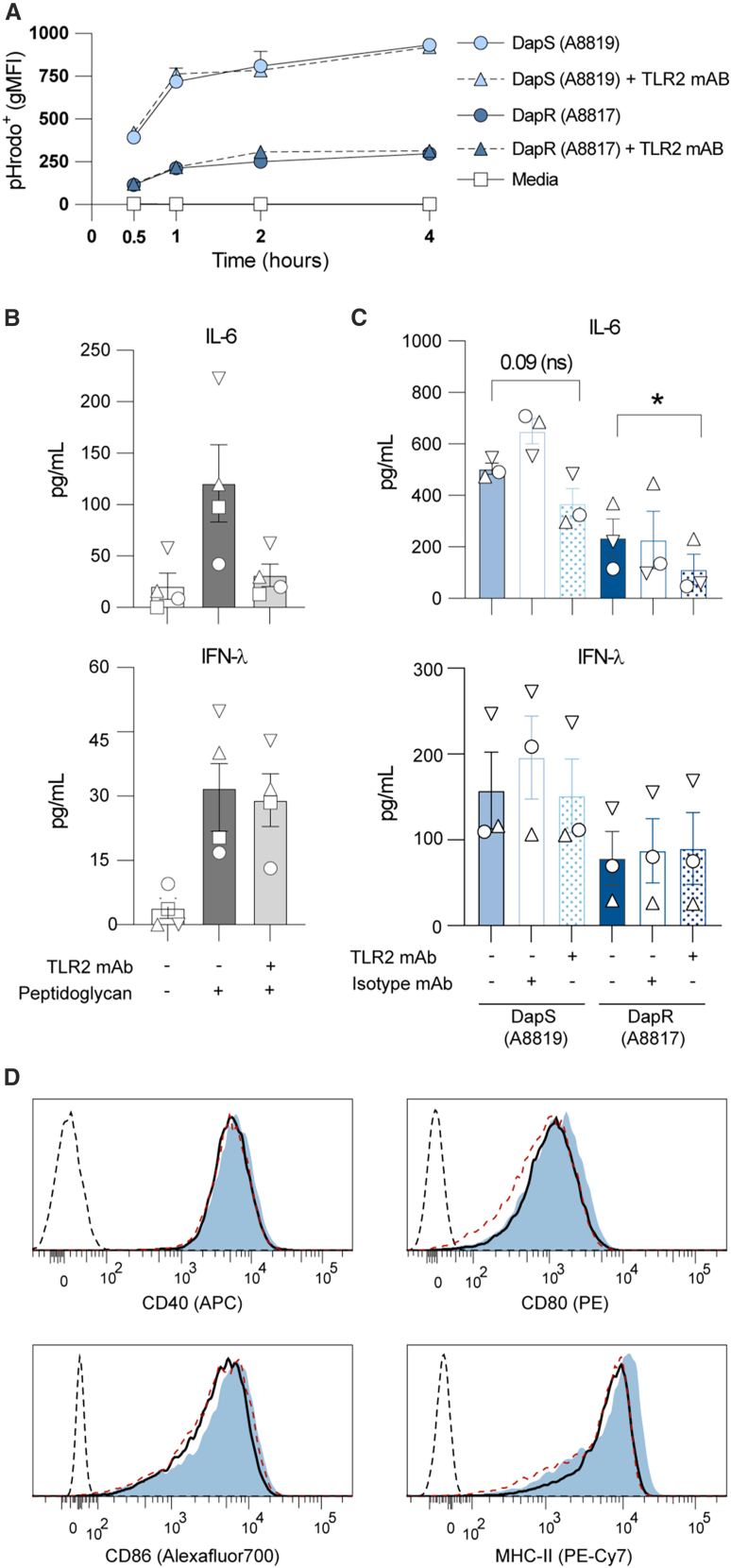
TLR2 is required for MutuDC sensing of, but not internalization of MRSA (A) Relative pHrodo labeled MRSA internalization by MutuDC over 4 h following stimulation with DapS A8819 (light blue symbols) or DapR A8817 (dark blue symbols), or media alone (white squares). Prior to stimulation, MutuDC were pre-treated for 1 h with TLR2 blocking antibody (clone T2.5; triangles with dashed lines) or media alone (circles with filled lines). Relative MRSA internalization by each DC subset is expressed as the gMFI of pHrodo. Results show the mean and (SD) of duplicates from one experiment, representative of two independent experiments. (B) Cytokine secretion (pg/mL) by MutuDC stimulated with TLR2 ligand peptidoglycan of *S. aureus* (PGN-SA) (10 μg/mL) or (C) DapS (A8819; light blue) or DapR (A8817; dark blue) MRSA (MOI of 10) for 18 h. MutuDC were first pre-treated with either TLR2 blocking antibody (dot-filled bars) or media alone (filled bars) as in A, or an isotype control (clone 163D3, empty bars) at 1 μg/mL. Results pooled from four (B) or three (C) independent experiments and expressed as the mean ± SEM, with each symbol (circle, square, and directional triangles) representing paired experimental replicates (*n* = 3). Statistical significance determined using paired *t* test and reported as indicated by an ∗ when *p* ≤ 0.05. (D) Expression of surface activation markers by MutuDC stimulated with DapS A8819 MRSA. DC were pre-treated with TLR2 blocking antibody (black trace), isotype control (dashed red trace), and media alone (light blue shaded). Unstained control sample is shown for each marker (black dashed trace). Data shown from one experiment, representative of three independent experiments.

TLR2 recognizes bacterial cell wall components, including peptidoglycan.^31^^,^^32^ Others have previously implicated TLR2 in the uptake and sensing of Staphylococcal strains in various cell types.^33^^,^^34^^,^^35^^,^^36^ We hypothesized that differences in cell wall composition in DapR MRSA^5^ may alter sensing by TLR2. To investigate this, we utilized the TLR2 blocking antibody clone T2.5. Blocking TLR2 on MutuDC, followed by incubation with the pHrodo labeled DapS (A8819) or DapR (A8817) clinical pair, had no effect on the internalization of either of these isolates of MRSA (Figure 3A). Of note, TLR2 blocking was effective as we observed inhibited secretion of IL-6 by DC in response to peptidoglycan (Figure 3B). Blocking of TLR2 with T2.5, but not the isotype control, slightly reduced the secretion of IL-6 by MutuDC following stimulation with DapS A8819 and significantly with DapR A8817 isolates (Figure 3C). While blocking TLR2 decreased the secretion of IL-6 by MutuDC stimulated with MRSA, it did not account for the differential in cytokine secretion elicited by this pair, as DC secreted less IL-6 in response to DapR A8817 as compared to DapS A8819 regardless of TLR2 blocking (Figure 3B).

TLR2 blocking had no impact on the secretion of IFN-λ by MutuDC following treatment with the A8819/A8817 isolates or the peptidoglycan control (Figure 3C). Notably, the commercial peptidoglycan control is probably contaminated with nucleic acids, as it induced low levels of TLR-2 independent IFN-λ. Similarly, the surface phenotype of MutuDC was unaffected by blocking with TLR2 antibody, with no differences in the expression of CD40, CD80, CD86, or MHC-II following stimulation with MRSA (Figure 3D).

Thus, TLR2 was dispensable for both MutuDC uptake of DapS or DapR MRSA and cell surface activation of MutuDC. However, it was involved in sensing both DapS and DapR MRSA, contributing to IL-6 production by MutuDC in response to both clinical isolates.

### Disruption of the MRSA cell envelope enhances DC responses

**Figure 4:**
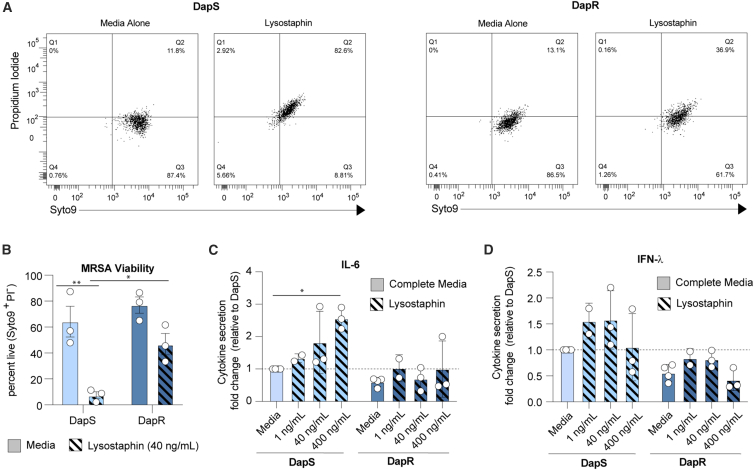
Lysostaphin treatment induces death and enhances DC response to DapS MRSA but has minor effects on viability and responses to DapR MRSA (A) Viability of DapS (A8819) MRSA or DapR (A8817) MRSA following 1 h incubation with complete media or lysostaphin (40 ng/mL). Data shown from one experiment representative of three independent experiments (*n* = 3). (B) Percentage of live (Syto9^+^PI^−^) DapS (A8819) MRSA or DapR (A8817) MRSA following 1h incubation with complete media or lysostaphin (40 ng/mL), as in A. Data show the mean and SEM from three independent experiments (*n* = 3). ∗*p* < 0.05 and ∗∗*p* < 0.005; two-way ANOVA. (C and D) IL-6 and IFN-λ production by MutuDC following overnight stimulation with DapS (A8819) MRSA or DapR (A8817) MRSA, treated with indicated concentrations of lysostaphin. Data are expressed as fold change relative to response to DapS (Media). Data show the mean, and SEM pooled from two to three independent experiments (*n* = 2 or 3). ∗*p* < 0.05, two-way ANOVA.

We observed that TLR2 responses did not differentiate between DC responses to DapS and DapR MRSA. We therefore questioned whether the increased complexity of the cell wall of DapR MRSA clinical isolates compared to DapS could directly lead to diminished responses. We hypothesized that upon phagocytosis, the complex cell wall of DapR MRSA may be harder for bactericidal enzymes within DC to penetrate, thus preventing the recognition of intracellular DAMPs, e.g., DNA. We therefore utilized lysostaphin, an anti-microbial enzyme with potent activity against the staphylococcal wall.^37^ While pre-treatment of both DapS and DapR with lysostaphin decreased the viability of these isolates relative to the media control by approximately 90% and 50%, respectively (Figures 4A and 4B), only the lysostaphin treatment of DapS was consistently capable of enhancing the secretion of IL-6 by MutuDC (Figure 4C). In contrast, IFN-λ secretion was boosted, albeit non-significantly, in response to both DapS and DapR isolates following treatment with low to moderate concentrations of lysostaphin (Figure 4D). Higher concentrations of lysostaphin (400 ng/mL) tended to impair the IFN-λ response by MutuDC (Figure 4D).

These data indicated that lysostaphin permeabilized the cell wall of DapS MRSA much more efficiently than that of DapR MRSA. Permeabilization appeared to reveal further PAMPs to the MutuDC, since lysostaphin-treated DapS MRSA, and, to a lesser extent, DapR MRSA, were more stimulatory than their untreated controls.

### cGAS/STING pathway inhibitors blunt DC responses to DapS MRSA

The enhanced IFN-λ production in response to both DapS and DapR MRSA treated with enzymes targeting the cell wall was intriguing. In previous studies, we have shown that cDC1 produces IFN-λ exclusively in response to either sensing of dsRNA species through TLR3^38^ or sensing of cytoplasmic DNA/CDNs through the cGAS/STING pathway.^39^ Considering that MRSA isolates can elicit the production of IFN-λ by MutuDC, we proposed that cGAS/STING sensing was the most likely candidate for differential sensing of DapS and DapR clinical isolates by DC.

Using the small molecule inhibitor of cGAS, Ru.521, we found that the robust production of IFN-λ by MutuDC in response to A8819 and the calf thymus (CT)DNA control was potently inhibited (Figure S4A). Moreover, inhibition of cGAS further impaired the secretion of pro-inflammatory cytokines IL-6 and TNF in response to DapS A8819, with little to no effects on the responses to DapR A8817 (Figure S4A). Similarly, treatment with the STING inhibitor, H151, resulted in decreased production of IFN-λ, IL-6, and TNF from DC following stimulation with DapS A8819 but not DapR A8817 (Figure S4B). The low levels of IFN-λ induced in response to another clinical DapS/DapR pair (A9754/A9757) were also inhibited by the cGAS inhibitor (Figure S4C).

In Figure 1, the DC maturation marker that was differentially expressed predominantly in response to DapS was CD86. Impairment of cGAS and STING signaling in MutuDC resulted in the decreased expression of CD86 following DapS MRSA stimulation of MutuDC (Figures S4C and S4D); but led to little to no differences in the expression of CD40, CD80, or MHC-II (Figures S4C and S4D).

Together, these data suggested that the disparate capacity of DC to recognize the DapS A8819/DapR A8817 clinical pair is mediated via differential cGAS/STING activation.

### cGAS/STING responses to *S. aureus* clinical isolates are reduced upon the acquisition of antibiotic resistance

The activation of STING leads to its relocation from the ER to the Golgi compartment, phosphorylation, transport to multi-vesicular bodies, ubiquitination, and degradation.^40^^,^^41^^,^^42^ We have previously observed robust activation of STING in DC in response to synthetic STING ligands, peaking at approximately 90 min.^39^

**Figure 5:**
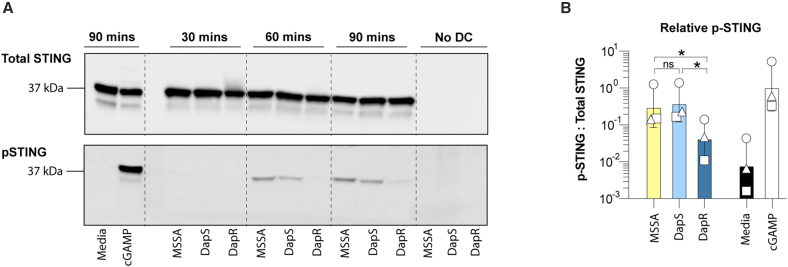
DapR MRSA fails to stimulate STING phosphorylation in DC (A) Western blot of cell lysates of MutuDC stimulated with complete media, 10 nmol 3′3′-cGAMP, MOI = 10 of MSSA (D85), DapS (A8819) MRSA, or DapR (A8817) MRSA, for the indicated times. (B) Quantitation of p-STING relative to total STING for stimulations as in “A,” at 90 min post stimulus. Bars show the geometric mean and geometric SD of replicates pooled from three independent experiments (*n* = 3). Statistical significance determined using paired *t* test and reported as indicated by ∗*p* ≤ 0.05.

We next cultured MutuDC with DapS and DapR strains of MRSA over a time course from 30 to 90 min and included a methicillin-sensitive *S. aureus* strain (MSSA) for comparison. We detected active, phosphorylated (p)STING as early as 1 h post stimulation with MSSA and DapS MRSA (Figure 5A). In contrast, pSTING was undetectable in MutuDC in response to DapR A8817 until a very weak signal at 90 min (Figure 5B). This indicates that STING activation is driving the sensing of MSSA and DapS MRSA by MutuDC, and that the differential, limited sensing of DapR MRSA by MutuDC is due to a lack of STING activation.

### DC production of 2′3′-cGAMP in response to DapR MRSA is blunted in comparison to their response to DapS MRSA

Our data indicate that DapR MRSA fails to activate cGAS/STING in DC. In response to bacteria, STING can be activated either indirectly by 2′3′-cGAMP produced by cGAS following bacterial DNA sensing, or directly via 3′3′-cGAMP produced by the bacteria.^43^

**Figure 6:**
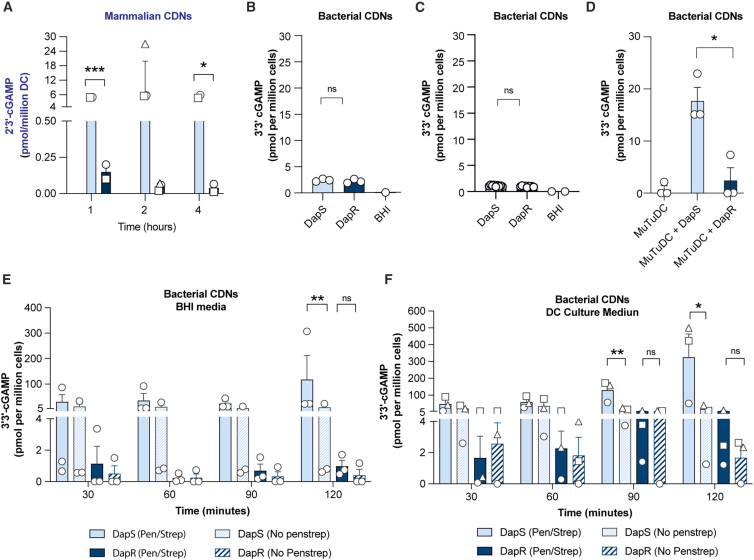
MRSA isolates differentially induce 2′3′-cGAMP production in DC and differentially secrete 3′3'-cGAMP in response to stress signals (A) Detection of mammalian 2′3′-cGAMP (pmol/million DC) in MutuDC (lysates) following 1, 2 or 4 h stimulation with DapS (A8819; light blue) MRSA or DapR (A8817; dark blue) MRSA at an MOI of 10. Bars show the mean and SEM of 2′3′-cGAMP detected from three biological replicates (*n* = 3). (B and C) Detection of bacterial 3′3′-cGAMP in fresh (B) or freeze-thawed (C) supernatants of DapS (A8819; light blue) MRSA or DapR (A8817; dark blue) MRSA cultured in brain heart infusion (BHI) medium overnight at a density of 4 × 10^8^ cfu/ml. (D) Detection of bacterial 3′3′-cGAMP in fresh MutuDC lysates following 2 h of stimulation with DapS (A8819; light blue) MRSA or DapR (A8817; dark blue) at an MOI of 10. Bars show the mean and SEM of 3′3′-cGAMP detected from three biological replicates (*n* = 3). (E and F) Bacterial 3′3′-cGAMP production by DapS (A8819; light blue) MRSA or DapR (A8817; dark blue) isolates cultured in BHI culture media (E) or DC culture media (F); with each media alone (solid bars) or supplemented with antibiotics (dashed bars), consisting of penicillin (10 units/ml) and streptomycin (10 μg/mL) and incubated overnight at a density of 8 × 10^6^ cfu/ml. Bars indicate 3′3′-cGAMP at 30, 60, 90, and 120 min post incubation, showing the mean and SEM from three biological replicates (*n* = 3). (A, B, C, and D) Significance reflective of an unpaired two-tailed *t* test using Holm Sidak correction, reported as indicated by ∗*p* ≤ 0.05 and ∗∗∗*p* < 0.0005. (E and F) Paired two-tailed *t* test where ∗*p* < 0.05 and ∗∗*p* < 0.005.

We first analyzed 2′3′-cGAMP production within DC, which is a direct readout of cGAS activity in response to cytosolic DNA. MutuDC produced significant levels of 2′3′-cGAMP in the presence of DapS MRSA but not in response to DapR MRSA (Figure 6A). Approximately 6–25 pmol of 2′3′-cGAMP was produced per 1 million DC in response to DapS MRSA, while DapR MRSA barely induced detectable 2′3′-cGAMP from DC. These data suggest that DNA is efficiently released from DapS MRSA but not from DapR MRSA due to alterations in the composition of the bacterial cell envelope. It is also possible that DNA damage in the DC (e.g., mitochondrial damage) may be stress-induced by phagocytosis of DapS MRSA, although this was not further investigated. Overall, these data are in line with our inhibitor studies (Figure S5) that strongly suggest a role for cGAS in the differential sensing of DNA from DapS and DapR MRSA, leading to differential activation levels of STING (Figures 5 and 6).

To investigate DapS and DapR produced bacterial CDNs, we measured the abundance of 3′3′-cGAMP in fresh (Figure 6B) or freeze-thawed (Figure 6C) bacterial supernatants from growth media [brain-heart infusion broth (BHI)]. We detected low levels of 3′3′-cGAMP from fresh culture broth of high concentrations of both DapS and DapR MRSA (Figure 6B), suggesting both isolates have the capacity for similar low-level production and export of CDNs. Notably, freeze-thawing of bacterial supernatants leads to the loss of detection of CDNs in the supernatant (Figure 6C).

Next, to determine whether DapS and DapR MRSA can also produce 3′3′-cGAMP production within acidic phagolysosomes of DC, we tested the lysates of MutuDC that had been incubated with either DapS or DapR MRSA. We observed 10 to 20-fold greater 3′3′-cGAMP isolated from lysates derived from MutuDC infected with DapS, as compared to those from MutuDC with DapR (Figure 6D).

Recently, others have shown that β-lactam antibiotics can induce the production of the CDN c-di-AMP from *S. aureus*.^44^ As our MutuDC cultures were carried out in media containing the common antibiotic cocktail, penicillin (a β-lactam antibiotic targeting the cell wall) and streptomycin, we therefore questioned whether MRSA produces 3′3′-cGAMP in this media as a result of antibiotic-induced stress. To address this, we tested DapS or DapR CDN production in response to antibiotic stress in both their growth media, BHI (Figure 6E), and DC culture medium (Figure 6F). After 2 h of culture, the presence of antibiotics in BHI induced increased 3′3′-cGAMP production by DapS MRSA, but not DapR MRSA. In DC culture medium, DapS MRSA produced enhanced levels of 3′3′-cGAMP by 1.5 h in the presence of antibiotics. By 2 h in DC culture media, the DapS MRSA produced up to 100-fold more 3′3′-cGAMP in the presence of antibiotics than in the absence of antibiotics. DapR MRSA produced 2- to 5-fold more 3′3′-cGAMP in the presence of antibiotics, but this level was only reached by DapS MRSA in the absence of antibiotics. Our results, therefore, indicate that DapS and DapR MRSA respond differently when exposed to stress conditions, with high levels of 3′3′-cGAMP produced only by DapS MRSA.

### STING mediates the differential sensing of DapS/DapR clinical isolates

**Figure 7:**
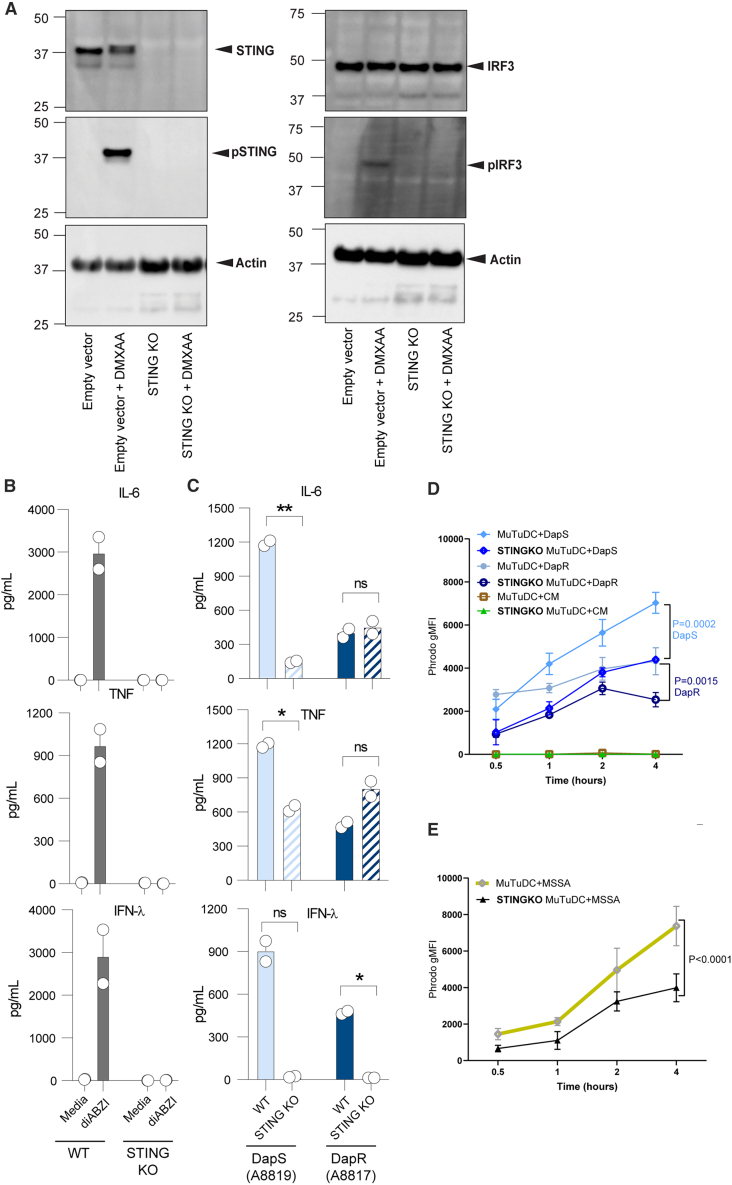
Differential recognition of MRSA by MutuDC is dependent on STING (A) Western blot for total STING, pSTING, IRF3, pIRF3, and actin in lysates of WT (Cas9-expressing) and STING^KO^ MutuDC, following 90 min treatment with the STING agonist DMXAA (10 μg/ml) or media alone. (B) IL-6, TNF, and IFN-λ production (pg/mL) by WT (solid bars) and STING^KO^ MutuDC (dashed bars) following overnight treatment with STING agonist diABZI (0.5 μM) or (C) DapS (A8819; light blue) or DapR (A8817; dark blue) MRSA. Data show the mean and range of replicates pooled from two independent experiments (*n* = 2). (D) STING^KO^ or WT MutuDC were incubated for 0.5 to 4 h with media alone or pHrodo -labeled *S. aureus* strains: DapS (8819) MRSA, DapR (8817) MRSA, or (E) MSSA (D85), as shown. MSSA is shown on a separate graph for clarity. Uptake is shown as mean and S.D. of pHrodo gMFI detected in the WT or STING^KO^ MutuDC in 3 independent experiments (*n* = 3). *p* values shown were calculated by two-way ANOVA, comparing WT versus STING^KO^ MutuDC uptake for each bacterial strain or media controls.

Given the differential production of CDNs between DapS and DapR clinical isolates (Figures 6D–6F), and commensurate MuTuDC STING phosphorylation following stimulations with these bacteria (Figure 5), we sought to confirm the role of STING in sensing these isolates. To this end, we utilized CRISPR/Cas9 gene editing to generate MutuDC deficient in STING (i.e., STING^KO^ MutuDC), which was confirmed at the protein level by immunoblot (Figure 7A). When compared to the empty vector control, STING^KO^ MutuDC had no detectable expression of STING, nor pSTING, nor phosphorylated Interferon Regulatory Factor-3 (pIRF3), in response to the murine STING agonist, DMXAA (Figure 7A). STING^KO^ MuTuDC were unable to produce any detectable cytokines following stimulation with a STING agonist (Figure 7B).

In response to the DapS isolate of MRSA, IL-6 and TNF cytokine responses of STING^KO^ MutuDC were significantly blunted, as compared to the parental wildtype control cells (Figure 7C). In contrast, there were no significant differences in the secretion of these cytokines between the wildtype and STING^KO^ MutuDC following stimulation with the DapR clinical isolate (Figure 7C). Intriguingly, the IFN-λ response was entirely dependent on STING for both treatments with the DapS and DapR isolates. Thus, the differential sensing of DapS and DapR MRSA by MutuDC is regulated at the level of STING signaling, where STING is absolutely required for the production of IFN-λ in response to both strains. The strong STING activation induced by DapS MRSA induces high IFN-λ, which is totally STING-dependent, and high TNF and IL-6, which are partially STING-dependent and also reliant on TLR2 (Figure 3). In response to DapR MRSA, we observe an overall blunted cytokine response, which is generated mostly by TLR2 signaling, with the blunted and variable in magnitude IFN-λ response totally dependent on STING signaling.

Since the uptake of DapR MRSA by MutuDC was poorer than DapS MRSA (Figure 2), we investigated whether blunted STING signaling could also play a role in these differences. We compared the uptake of pHrodo -labelled DapS (8819) and DapR (8817) strains by the wild-type and STING^KO^ MutuDC. Consistent with our previous data (Figure 2), Dap R MRSA was less efficiently phagocytosed than DapS MRSA by wild-type MutuDC (Figure 7D). However, the uptake of DapS MRSA was decreased in STING^KO^ MutuDC; to a level resembling the uptake of DapR MRSA in the wildtype cells (Figure 7D). It should also be noted that although DC phagocytosed DapR MRSA relatively poorly, the uptake was further decreased in the STING^KO^ cells (Figure 7D). The uptake of MSSA was also examined for comparison, similarly, the uptake of MSSA was blunted in the STING^KO^ cells (Figure 7E). Thus, STING signaling is required for optimal uptake of *S. aureus* strains by DC.

## Discussion

Previously, we have reported that single-nucleotide mutations acquired by MRSA isolates during infection and conferring Dap resistance can fundamentally change the extent to which these DapR isolates are able to activate FLDC.^10^ Here, we confirm these findings in primary DC, and demonstrate that a DapR isolate carrying these mutations is internalized by DC at significantly lower rates than the DapS parent isolate. Moreover, we identify that the differential sensing of these isolates by DC is regulated in part by TLR2 at the cell surface, and importantly, by the intracellular sensors cGAS and STING.

We developed a flow-based method to quantify the internalization of S. aureus clinical isolates stained with the pH-sensitive dye pHrodo, and subsequently validated this model using conventional EM. Using this approach, we demonstrate that both primary cDC1, cDC2, and MutuDC internalize the DapR A8817 isolate at a significantly lower rate than its DapS A8819 parent strain. Of note, EM imaging of cDC1 demonstrated clinical isolates both in endosomal structures and apparently “free” in the cytoplasm, commensurate with an established capacity to evade endosomal destruction and escape into the cytosol.^45^^,^^46^ Although TLRs are not phagocytic receptors, they are capable of co-operating with phagocytic receptors to enhance internalization.^47^ However, despite TLR2 sensing contributing to cytokine production in response to both isolates, we found no difference in the internalization of these isolates after blocking TLR2, suggesting uptake occurs in a TLR2-independent manner. Instead, we have shown that STING signaling is required for optimal uptake of both MRSA and MSSA strains (Figures 7D and 7E).

Our results suggest that PRR ligand availability modulates the sensing of DapS and DapR isolates. For DapS, lysostaphin treatment, which increased the permeability of the cell membrane, led to increased secretion of IL-6 and IFN-λ by MutuDC in a dose-dependent manner. This had minimal effect on DapR isolates. Others have previously shown that only live lab strains of *S. aureus*, and not heat or UV-inactivated strains, were capable of stimulating the cGAS/STING pathway in human monocyte-derived DC.^48^ Similar results have also been reported in murine macrophages.^22^ These collective observations lead us to propose that within the phagolysosome of cDC1, only DapS can be effectively lysed to release DNA for cGAS/STING detection. We proposed that the complex outer membrane of DapR was difficult to degrade, failing to release DNA. Indeed, blocking cGAS/STING with commercial inhibitors had a profound impact on DC surface activation and cytokine secretion in response to DapS isolates, with the impaired upregulation of both surface CD86 and secretion of IL-6 and TNF. Moreover, the production of IFN-λ by MutuDC in response to DapS was reduced in the presence of STING or cGAS inhibitors and further in the complete knockout of STING, which resulted in the ablation of IFN-λ secretion by MutuDC in response to both DapS and DapR isolates. We further observed poor activation of STING by DapR MRSA. We therefore investigated whether the DNA sensor cGAS directly senses genomic DNA from the DapS and DapR strains to produce mammalian 2′3′-cGAMP. We observed MutuDC production of 2′3′-cGAMP in response to DapS MRSA, at levels at least 30-fold higher than produced in response to DapR MRSA (Figure 6A). Our result thus suggests that DNA is being released from DapS MRSA and translocated to the cytoplasm to activate cGAS.^49^ The poor response to DapR MRSA is likely attributable to a combination of the reduced ability to phagocytose bacteria, which we show is at least partially dependent on STING signaling (Figures 7D and 7E), and difficulty in the release of bacterial DNA within the phagosome due to the altered cell envelope of DapR MRSA.

Staphylococcol strains, such as many bacteria, express cyclic oligonucleotide-based antiphage signaling systems (CBASSs). The CBASS system, similar to eukaryotic cGAS/STING, consists of 2 subunits: a cGAS-like cyclic nucleotidyltransferase enzyme that produces cyclic nucleotides upon sensing bacteriophage infection, and an effector protein that binds the CDNs.^50^ In the case of bacteria, binding of CDNs has the effect of triggering death or growth arrest of the bacterial host.^25^^,^^51^ Banh and colleagues recently showed the ability of staphylococcal CBASS to, surprisingly, recognize phage RNA, leading to triggering of the bacterial CBASS-dependent immune response, including membrane disruption, without lysis.^52^ 3′3′-cGAMP CDN is uniquely produced by bacteria through the CBASS system and is a danger signal recognized directly by mammalian STING. Thus, production of 3′3′-cGAMP by MRSA could potentially act as both a stimulus for STING in DC, as well as a means of disrupting the bacterial membrane, enabling more effective degradation in the phagolysosome, increased DNA release, and potential activation of mammalian cGAS.

It has been demonstrated that some bacterial cGAS-like proteins are constitutively active *in vitro*^53^*.* Indeed, our data indicated that 3′3′-cGAMP is produced at very low concentrations by both DapS and DapR MRSA in their broth culture supernatant, in the absence of any DC. In culture with MutuDC, we detected 3′3′-cGAMP produced by DapS MRSA in MutuDC lysates, at least 6- to 10-fold higher than detected in MutuDC lysates from DapR/MutuDC cultures. Surprisingly to us, in media containing penicillin and streptomycin antibiotics, the DapS and DapR MRSA both produced 3′3′-cGAMP, with DapS MRSA producing at least 20–60 -fold higher levels than DapR MRSA. Thus, we propose that due to differences in the complexity of the cell envelope, DapS, but not DapR, MRSA produce CDNs as a means to prolong survival in the presence of antibiotics and/or the low pH endosome of MutuDC. The altered cell envelope of DapR MRSA acts as protection against not only the antibiotic Daptomycin, but also other cell stresses and, in so doing, evades mammalian innate immune sensing, including bacterial uptake, through the cGAS/STING pathway.

Several studies have focused on bacterial-derived CDNs because of their capacity to directly activate the STING signaling pathway. Rosen and colleagues showed that the activation of STING by CDN attenuated *Klebsiella pneumonia* virulence in the mouse lung.^54^ The immunostimulatory properties of CDNs as novel immunotherapeutics have been observed to induce robust innate immune responses and enhanced host resistance against infection associated with MRSA.^55^ However, *S. aureus* can regulate its antibiotic resistance phenotype by altering CDN secretion. A study on MRSA strains demonstrated that CDN (c-di-AMP) can alter methicillin resistance levels of the bacteria by switching their levels of production.^56^ Recently, a striking consequence of community-acquired (CA)-MRSA lacking LTA polymer in the cell wall was observed. Drastic increases in the intracellular levels of the CDN c-di-AMP were produced as a consequence of cell wall changes, consequently helping bacteria to cope with extreme cell wall stress, in addition to controlling the cell size of *S. aureus*.^26^ Indeed, there have been numerous reports of both c-di-GMP or c-di-AMP production leading to or enhancing antimicrobial resistance.^57^ Although in our study, we have not explored the production of c-di-GMP or c-di-AMP by DapS and DapR MRSA, we show that 3′3′-cGAMP production is altered in MRSA bacterial stress conditions, including the presence of β-lactam antibiotics. However, intriguingly, the altered cell envelope of DapR MRSA protected the bacteria from extracellular stress conditions, which resulted in low production of 3′3′-cGAMP. Considering that the only differences between DapS and DapR strains A8817/A8819 are mutations in 5 genes, including *cls2* and *mprf* genes^5^ and that we have previously shown that these genes contribute to the differences in sensing of DapS and DapR by DC,^10^ our data suggest that these and other common mutations in *cls2* and *mprF* in DapR isolates, regulating membrane phospholipid biosynthesis,^5^ are sufficient to modulate CDN secretion by *S. aureus* and in turn, the mammalian host immune response.

Subversion of host signaling molecules, particularly CDNs, has emerged as a common evolutionary strategy of pathogens to counteract host innate immune responses.^58^ It has been observed that human immunodeficiency viruses (HIVs), simian immunodeficiency viruses (SIVs), or murine leukemia virus (MLV) obviate cytoplasmic surveillance pathway responses by concealing viral nucleic acids within capsid structures and/or limiting the accumulation of cytosolic viral DNA.^59^ Similar to this, our findings on CDN production by murine DC suggested that the thickened and complex cell wall and membrane of DapR MRSA reduce the capacity of DapR isolates to induce CDN production and, as a consequence, their nucleic acids remain concealed. Taken together, our findings indicated that the mutations in the A8817 DapR strain, most likely in *mprF* and *cls2,* altered the immunogenicity of the A8819/A8817 clinical pair by regulating the production and secretion of CDN. An elegant study recently showed that the antibiotic-mediated disruption of thymidine synthesis promotes elevated levels of the bacterial second messenger cyclic di-AMP (c-di-AMP) in *S. aureus*, consequently inducing host STING activation and inflammation.^44^ This group also noted increased c-di-AMP in response to β-lactam antibiotics. There is some concern that elevated STING activation may induce excessive elevation and lung pathology in infection;^44^ however, *in vivo* studies in STING-deficient mice resulted in increased mortality, more bacterial burden in the lungs, and elevated inflammation during pulmonary *S. aureus* infection.^20^ Thus, perhaps a “goldilocks” effect is required, where enough bacterial CDN response can alert the immune system, without evoking excessive inflammation. Here, we have further identified that IFN-λ is produced through STING activation in DC upon sensing MRSA. It is tempting to speculate a role of IFN-λ in controlling bacterial infection *in vivo*, this requires further examination.

**Figure 8:**
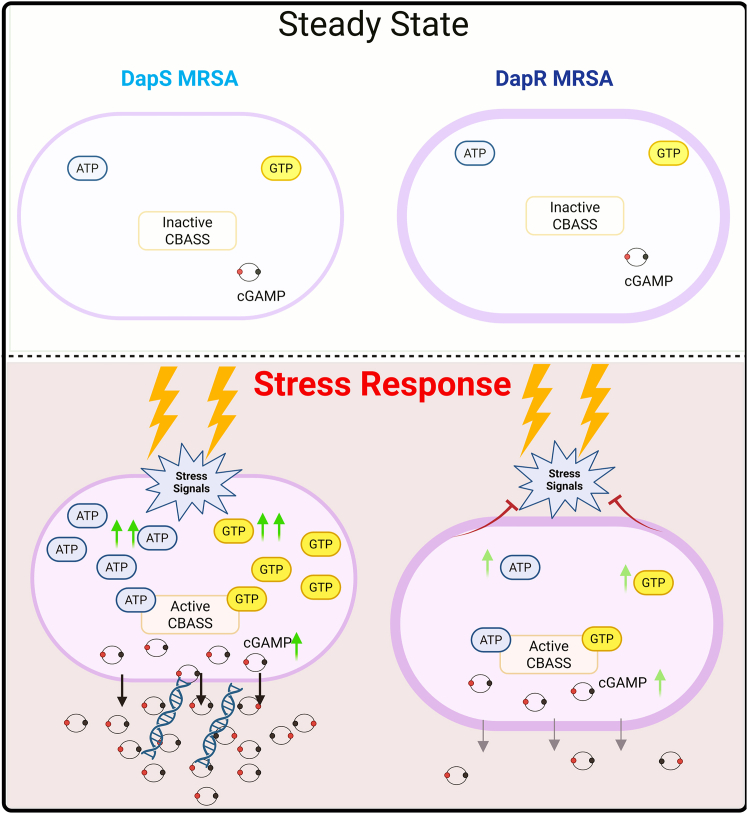
CDN secretion in response to stress by DapS, but not DapR, MRSA In steady state, both DapS and DapR MRSA produce very low levels of 3′3′- CDN. Under stress conditions, bacteria can activate the CBASS defense system, triggering the production of the bacterial CDN 3′3′-cGAMP, a potent mammalian STING agonist. Under stress, DapS MRSA produces plentiful 3′3′-cGAMP, and the weakened bacterial envelope can lead to bacterial DNA release, providing mammalian cGAS stimulus and 2′3′-cGAMP production and further STING activation, which also enhances phagocytosis. The complex cell wall of DapR MRSA provides “innate” protection during stress and prevents the triggering of CBASS to produce CDN.

Taken together, our observations suggest a model in which the CBASS defense system of DapS MRSA strongly senses extracellular stress, which triggers the bacterial production of 3′3′-cGAMP, aiding in weakening of the bacterial cell wall (Figure 8). The bacterial DNA released through the enzymatic digestion of MRSA in the phagolysosome of DC leads to cGAS activation and production of 2′3′-cGAMP and, together with 3′3′-cGAMP, subsequent activation of STING signaling to induce enhanced phagocytosis of the bacteria and increased IFN-λ and cytokine responses by DC.

### Limitations of the study

As our study was carried out *in vitro*, future *in vivo* experiments to clarify the role of STING, IFN-λ, and indeed the CBASS system of S. aureus and other bacterial strains in inducing and optimizing innate immunity to infection, will lend further insight into this work. We were limited in this study by just examining murine DC populations; a comprehensive analysis of the responses of human DC and other myeloid cell types to *S. aureus* strains is required. These will clarify whether antagonists of the STING pathway may be useful therapeutics for hard-to-clear bacterial infections and whether IFN-λ may also be useful in the treatment of bacterial infections.

## Resource availability

### Lead contact

• Requests for further information and resources should be directed to and will be fulfilled by the lead contact, Meredith O’Keeffe (meredith.okeeffe@monash.edu).

### Materials availability

• All unique reagents generated in this study are available from the lead contact with a completed materials transfer agreement.

### Data and code availability

• Original western blot images are in the supplementary data. Flow cytometry and microscopy data reported in this paper will be shared by the lead contact upon request.
• This paper does not report original code.
• Any additional information required to reanalyze the data reported in this paper is available from the lead contact upon request.

## Acknowledgments

We thank Andrew Fryga and the team at Monash FlowCore for their expertise in performing FACS sorting for these experiments. We also thank the staff at the Monash Animal Research Platform for maintaining the mouse colonies required for these experiments. M. O’Keeffe was supported by a 10.13039/501100000925National Health and Medical Research Council (10.13039/501100000925NHMRC) Senior Research Fellowship (APP1077633) and ARC Discovery Project (DP210103122). D. De Nardo was supported in part by an 10.13039/501100020481Australian and New Zealand Society for Immunology (ASI) Breakthrough Immunology Award. T. Patton, E. S. Pang, R. Venkatraman, and A. Shoppee were supported by Australian Government Research Training Program (RTP) Scholarships. K. Balka was supported by a Monash Silver Jubilee Postgraduate Research Scholarship and a Monash Graduate Excellence Scholarship. Nazneen Jahan was co-funded by a Monash Graduate Scholarship and Faculty International Tuition Scholarship. A. Y. Peleg was funded by an Australian National Health and Medical Research Council Practitioner Fellowship (APP1117940) and project grants (APP1047918, APP1144303, and APP2002921). The CASS Foundation (grant 7319: M. O’Keeffe, R. Lundie, T. Patton, and A. Y. Peleg) also supported this work.

## Author contributions

T.P. and N.J.: investigation, methodology, formal analysis, visualization, and writing – original draft; J.-H.J.: methodology, investigation, and resources; X.K.: methodology and investigation; E.S.P. and R.L.: investigation, methodology, and supervision; K.B. and R.V.: investigation and methodology; V.O. and J.C.: investigation and methodology; S.A.: investigation; P.T.: methodology and investigation; A.S.: methodology and investigation; G.R.: resources and methodology; A.P., D.D.N., and M.O.K.: conceptualization, methodology, formal analysis, funding acquisition, project administration, supervision, and writing – original draft. All authors contributed to writing-review and editing.

## Declaration of interests

ESP is an employee and shareholder of CSL Ltd. All other authors declare no competing interests.

## Declaration of generative AI and AI-assisted technologies in the writing process

Generative AI and AI-assisted technologies were not used in this work.

## STAR★Methods

### Key resources table

**Table:** 

REAGENT or RESOURCE	SOURCE	IDENTIFIER
**Antibodies**		

Biotin anti-CD317	Purified from hybridoma in house	Clone 120G.8
APC- anti-CD40	BD Biosciences	Clone 3/23, Cat: 558695; RRID: AB_1645224
FITC anti-CD49b	BD Biosciences	clone DX5, Cat: 553857; RRID:AB_395093
PE- anti-CD80	BD Biosciences	clone 16-10A1, Cat: 561955; RRID: AB_10892805
BV650- anti-CD80	BD Biosciences	clone 16-10A1, Cat: 563687; RRID: AB_2738376
AlexFluor700- anti-CD86	BD Biosciences	clone: GL1, Cat: 560581; RRID: AB_1727517
APC- anti-PD-L2 (CD273)	BD Biosciences	clone TY25, Cat:560086; RRID: AB_1645223
BUV 395- anti-CD172	BioLegend	clone P84, Cat: 740282; RRID:AB_2740021
BV 421- anti-CD11c	BioLegend	clone N418, Cat: 117330; RRID:AB_11219593
BV650- anti-CD8a	BioLegend	clone 53–6.7, Cat: 100742; RRID:AB_2563056
FITC- anti-CD161c	BioLegend	clone PK136, Cat: 553164 RRID:AB_394676
AlexFluor488- anti-CD3	BioLegend	Clone 17A2, Cat: 100212; RRID: AB_493530
PE-Cy7- anti-CD86	BioLegend	Clone GL-1 Cat:; RRID: AB_439783
APC-Cy7 anti-MHC-II (clone M5/114.15.2; BioLegend)	BioLegend	Clone M5/114 Cat: 107628,; RRID: AB_2069377
PE-Cy7-*anti*-MHC-II (clone M5/114.15.2; BioLegend).	BioLegend	Clone M5/114, Cat: 107630; RRID: AB_2069376
BV605- anti-PD-L1 (CD274)	BioLegend	clone 10F.9G2, Cat: 124321; RRID: AB_2563635
mAB mTLR2- anti-mouse/human TLR2	InvivoGen	Cat# mab-mtlr2; RRID:AB_763722
Iso-control for mAB mTLR2 (mouse anti-human CD14; IgG1, kappa)	Purified from hybridoma in house	Clone 63D3, Cat# 19781; RRID:AB_2737062
rabbit anti-phospho-STING (Ser366) (clone D7C3S, Cell Signaling Technology),	Cell Signaling Technology	Cat# 13647; RRID:AB_2732796
rabbit anti-STING (clone D2P2F, Cell Signaling Technology),	Cell Signaling Technology	Cat# 4302; RRID:AB_1904036
rabbit anti-IRF3 (clone D83B9, Cell Signaling Technology),	Cell Signaling Technology	Cat: 79945; RRID: AB_2799943
rabbit-anti phospho IRF3 (E6F7Q, Cell Signaling Technology)	Cell Signaling Technology	Clone C4, Cat# sc-47778
Mouse monoclonal anti-beta ACTIN HRP antibody	Santa Cruz Biotechnology	HRP; RRID:AB_2714189
Anti-IL6 monoclonal antibody (ELISA capture)	BD Biosciences	Clone MP5-20F3, Cat#: 554400; RRID:AB_398549
Anti-IL-6 monoclonal antibody, Biotin (ELISA detection)	BD Biosciences	Clone MP5-32C11, Cat# 554402; RRID:AB_395368
Anti-TNF Polyclonal Antibody (ELISA capture)	PeproTech (ThermoFisher Scientific)	Cat# 500-P64-50ug; RRID:AB_147649
TNF alpha Polyclonal Antibody, Biotin, (ELISA detection)	PeproTech (ThermoFisher Scientific)	Cat# 500-P64bt-25ug; RRID:AB_147650
Mouse IL-28A/B (IFN-lambda 2/3) Antibody	R & D Systems	Cat# MAB17892; RRID:AB_2125204
Mouse IL-28A/B (IFN-lambda 2/3) Biotinylated Antibody	R & D Systems	Cat# BAM17891; RRID:AB_2125206

**Secondary reagents for flow or western blotting**		

PE-Cyanine7- (PE-Cy7) streptavidin	BD Biosciences	Cat #: 557598
HRP conjugated goat Anti-rabbit IgG	Cell signaling Technology	Cat #: 7074

**Bacterial and virus strains**		

MRSA: 8819 (DapS)	First described in doi: https://doi.org/10.1371/journal.pone.0028316	N/A
MRSA: 8817 (DapR)	doi: https://doi.org/10.1371/journal.pone.0028316	N/A
MRSA: A9754 (DapS)	doi: https://doi.org/10.1371/journal.pone.0028316	N/A
MRSA: A9757 (DapR)	doi: https://doi.org/10.1371/journal.pone.0028316	N/A
MRSA: A9763 (DapS)	doi: https://doi.org/10.1371/journal.pone.0028316	N/A
MRSA: A9764 (DapR)	doi: https://doi.org/10.1371/journal.pone.0028316	N/A

**Chemicals, peptides, and recombinant proteins**		

LyoVec™, used at 10% v/v	InvivoGen	Cat #: lyec-1
Lipofectamine 2000 Transfection Reagent	Thermo Fisher Scientific	Cat# 11668030
Ru.521 (used at 5 μg/ml), Mouse cGAS inhibitor	InvivoGen	Cat#: inh-ru521-2
H-151 (used at 500 ng/mL), STING inhibitor	InvivoGen	Cat #: inh-h151
2′3′-cGAMP - Cyclic [G(2′,5′)pA(3′,5′)p]	InvivoGen	Cat# tlrl-nacga23
3′3′-cGAMP- Cyclic [G(3′,5′)pA(3′,5′)p]	InvivoGen	Cat#: tlrl-nacga
SA-PGN (peptidoglycan from *Staphylococcus aureus*)	InvivoGen	Cat#: tlrl-pgns2
diABZI (diABZI (compound 3) trihydrochloride)	InvivoGen	Cat#: tlrl-diabzi-2 CAS#: 2138299-34-8
DMXAA (5,6-Dimethylxanthenone-4-acetic acid)	InvivoGen	Cat# tlrl-dmx
Mouse IL-6 (used as ELISA standard)	BD Biosciences	Cat#: 554582
Mouse TNF (used as ELISA standard)	PeproTech (ThermoFisher Scientific)	Cat#: 29-8321-65
Mouse IL28B (IFNlambda3; used as ELISA standard)	R & D Systems	Cat#: 1789-ML-025

**Critical commercial assays**		

DetectX® 3′,3′-Cyclic GAMP (cGAMP) Immunoassay Kit	Arbor Assay^TM^, distributed through Bioscientific Pty Ltd	1 Plate Kit Catalog Number K073-H1 5 Plate Kit Catalog Number K073-H5
2′3′-cGAMP Immunoassay Kit	Cayman Chemicals, distributed through Sapphire Biosciences Australia	Cat #: 501700
pHrodo™ Red, succinimidyl ester (pHrodo™ Red, SE)	ThermoFisher Scientific	Cat#: P36600
LIVE/DEAD™ BacLight™ Bacterial Viability Kit	Invitrogen, ThermoFisher Scientific	Cat#: L13152
Lumikine bioluminescent ELISA kit for IFN-α	InvivoGen	Cat#: luex-mifnav2

**Experimental models: Cell lines**		

Mutu DC Line 2114: derived from CD11c:SV40LgT C57BL/6 mice	Originally from Prof Hans Acha-orbea, Department of Biochemistry, University of Lausanne, Switzerland. Published here: https://doi.org/10.1182/blood-2007-06-097576	N/A
Mutu DC Line 2114 expressing Cas9	This paper	N/A
Mutu DC Line 2114 STING −/−	This paper	N/A
Human embryonic kidney (HEK) 293T cells	ATCC	CRL-3216

**Experimental models: Organisms/strains**		

C57BL/6 mice	Bred in house at Monash University Animal Research Platform	N/A

**Oligonucleotides**		

sgRNA targeting *sting* (CAGTTGGATGTTTGGCCTTC)	This paper	N/A

**Recombinant DNA**		

pALC2084: pALC2073 with *gfp* cloned into the EcoRI site	Published here: https://doi.org/10.1073/pnas.181206611	N/A
pRSV-Rev	Addgene	Plasmid# 12253
pMDLg/pRRE	Addgene	Plasmid# 12251
pMD2.G	Addgene	Plasmid# 12259
pFgH1tUT-GFP	Addgene	Plasmid# 70183
Lenti-Cas9-2A-Blast	Addgene	Plasmid#: 73310
pXPR_BRD003	Broad Institute, USA.	https://portals.broadinstitute.org/gpp/public/vector/details?vector=pXPR_003

**Software and algorithms**		

GraphPad Prism	http://www.graphpad.com/	RRID:SCR_002798
Adobe Illustrator	http://www.adobe.com/products/illustrator.html	RRID:SCR_010279
Image Lab	Bio-Rad Laboratories	RRID:SCR_014210
Adobe Illustrator	http://www.adobe.com/products/illustrator.html	RRID:SCR_010279

### Experimental model and study participant details

#### Mice and splenic DC

Male C57BL/6J mice at 6–12 weeks of age were obtained from the Monash Animal Research Platform (Melbourne, VIC, Australia) and handled in accordance with the Monash University Animal Ethics Committee (ethics number MARP/2016/027). Splenic DC were isolated as previously described.^39^

#### MutuDC lines

Murine tumor DC (MutuDC; line 2114) are derived from splenic tumors isolated from transgenic CD11c:SV40LgT C57BL/6 mice.^60^ MutuDC (line 2114, obtained from Tom Brodnicki at St. Vincent’s Hospital Melbourne) were seeded at 1.5 × 10^6^ cells per mL in complete media (CM; Gibco RPMI-1640 GlutaMAX, Thermo Fisher Scientific, Waltham, MA, USA) supplemented with 10% fetal calf serum (FCS; *In Vitro* Technologies, Noble Park, Melbourne, VIC, Australia), 100 μM 2-mercaptoethanol (Thermo Fisher Scientific) and 0.01% (w/v) penicillin/streptomycin (Thermo Fisher Scientific) adjusted to mouse tonicity (308 mOsm/L), and maintained at 37°C 10% CO_2_, with passaging at 80–90% confluency.

MutuDC were routinely tested for Mycoplasma contamination and tested negative.

#### Preparation of bacterial isolates and DC co-cultures

MSSA and paired clinical isolates of MRSA are as previously described,^10^ details of MRSA isolates utilised are listed in Table 1. Bacterial strains were grown in Brain Heart Infusion (BHI) broth overnight and the bacterial density was adjusted to 4 x 10^9^ CFU/mL, which was estimated by optical density of 600 nm (OD_600_). The bacterial cell suspension was serially diluted and plated on BHI agar to confirm the desired bacterial cell density was achieved.

**Table 1:** MRSA strains used in this study

MRSA Strain^10^	Daptomycin MIC (μg/mL)^a^	Genomic Mutations^b^	Mutated genes affecting cell wall or membrane
8819 (DapS)	0.25		
8817 (DapR)	2	5	*Mprf, cls2*
A9754 (DapS)	0.5		
A9757 (DapR)	4	9	*Mprf*
A9763 (DapS)	0.5		
A9764 (DapR)	4	3	*Mprf, cls2*

a Daptomycin resistance is defined as a minimum inhibitory concentration (MIC) > 1 μg/mL.

b Total number of point mutations observed in the clinical daptomycin-resistant daughter isolate with reference to the susceptible parent strain.

### Method details

#### Bacterial and DC co-cultures

DC were co-cultured with bacterial isolates for 18 hours in a humidified atmosphere at 37°C in 5% CO_2_ in CM at an equivalent MOI of 10 unless otherwise indicated. Tissue culture supernatants were collected to assess inflammatory cytokine secretion at endpoint, and cells analyzed by flow cytometry.

#### Preparation of bacteria for internalisation assays

Clinical bacterial isolates were transformed with pALC2084 expressing recombinant enhanced green fluorescent protein (eGFP) under the xyl/tetO promoter as described previously.^11^^,^^61^ Bacteria were cultured to induce eGFP expression in the presence of 1 μg/mL anhydrotetracycline (Atc) (Merck) for 5 hours. DC were stimulated with these eGFP recombinant strains as indicated, with the addition of Atc to the culture medium at a concentration of 1 μg/mL, as previously described.^11^

Where indicated, both GFP recombinants and their respective wild-type parental strains, were stained with the pH sensitive and amine reactive dye, pHrodo Red succinimidyl ester (Life Technologies, ThermoFisher Scientific), as per manufacturer’s instructions.

#### FACS sorting and flow cytometry

Enriched splenic DC were stained for FACS sorting into cDC1, cDC2 and pDC subpopulations (Figure S1) with the following antibodies: Brilliant Ultra Violet (BUV) 395- anti-CD172 (clone P84, Becton Dickinson), Brilliant Violet (BV) 421- anti-CD11c (clone N418, BioLegend, San Diego, USA), BV650- anti-CD8α (clone 53–6.7, Becton Dickinson), Biotin anti-CD317 (clone 120G.8, in house), PE-Cyanine7- (PE-Cy7) streptavidin (BD Biosciences), Fluorescein isothiocyanate- (FITC) anti-CD49b (clone DX5, BD Biosciences), FITC- anti-CD161c (clone PK136, BioLegend) and AlexFluor488-anti-CD3 (clone 17A2, BioLegend).

Following bacterial stimulation, spleen or MutuDC were stained with the following antibodies as appropriate: BV605- anti-PD-L1 (clone 10F.9G2; BD Biosciences), PE-anti-CD80 (clone 16-10A1; BD Biosciences), BV650- anti-CD80 (clone 16-10A1; BD Biosciences), PE-Cy7-anti-CD86 (clone GL1; BioLegend), AlexFluor700-anti-CD86 (clone GL1; BD Biosciences), APC- anti-PD-L2 (clone TY25; BD Biosciences), APC- anti-CD40 (clone 3/23; BD Biosciences) and APC-Cy7 anti-MHC-II (clone M5/114.15.2; BioLegend), PE-Cy7-*anti*-MHC-II (clone M5/114.15.2; BioLegend). Staining for both FACS sorting, and flow cytometry was performed in FACS buffer (2% FCS, 2 mM EDTA in PBS) for 30 min on ice. Viability was quantified via differential staining in propidium iodide (Sigma, Rowville, Australia). Cell sorting and analyses were performed on a BD Influx and BD Fortessa (BD Biosciences), respectively.

#### Internalisation assays

DC (total spleen DC or MutuDC) were incubated in a humidified incubator at 37°C, 5% CO_2_, stimulating for the indicated time periods with clinical bacterial isolates (MOI of 10) expressing recombinant enhanced green fluorescent protein (GFP), or wild-type isolates labeled with pHrodo. Splenic DC were pre-stained with antibodies for subset surface markers prior to commencing the assay. At the indicated time points, samples were immediately placed on ice, washed into ice-cold FACS buffer and analyzed via flow cytometry.

To investigate the role of TLR2 in internalisation, MutuDC were pre-incubated with TLR2 antibody MAB TLR2 (InvivoGen) or isotype control Ab (Clone 63D3, anti-human CD14) at 1ug/ml for 1 hr, prior to exposure to bacteria.

#### Conventional transmission electron microscopy

Samples were prepared after MutuDC/bacterial internalisation assays in 1.5 mL Eppendorf tubes and fixed for 2 h at room temperature in ruthenium red fixative (500 μg/mL ruthenium red, 1.2% [v/v] glutaraldehyde in 67 mM cacodylate buffer), rinsed, and post-fixed in ruthenium red post-fixative (500 μg/mL ruthenium red, 0.67% [w/v] OsO4, in 67 mM cacodylate buffer) for 30 min in the dark at room temperature. Cells were embedded in 4% (w/v) low melting point agarose for support. Dehydration was performed with ethanol and propylene oxide. Blocks of cells in agarose were embedded in Epon 812. Ultrathin sections of 70 nm were cut using a diamond knife (Ultra 45° Diatome) on a Leica Ultracut UCT7, placed on 50 mesh copper grids with carbon coated formvar support film and stained with 1% aqueous uranyl acetate and Waltons lead citrate. High resolution EM imaging was performed on a Jeol1400Flash TEM at 80 KeV.

#### cGAS and STING inhibition of MutuDC with Ru.521 and H-151

MutuDC were prepared at a concentration of 2×10^6^ cells/mL and incubated with Ru.521 (InvivoGen; 5 μg/ml) or H-151 (InvivoGen; 500 ng/mL) in LyoVec (10% [v/v] final concentration) for 1 hr at 37°C with 10% CO2. Cells were then stimulated overnight with staphylococcal isolates at a MOI of 10, in a humidified incubator at 37°C with 10% CO_2_.

Concentrations of the inhibitors were previously optimised through titration on MutuDC, selecting concentrations that enabled inhibition but maintained cell viability.

#### Cytokine assays

CCL1, CXCL1, CXCL10, IL-1β, IFN-β, IL-10 and IL-12p70 levels were quantified from either fresh or thawed tissue culture supernatants using a custom LEGENDplex (BioLegend) flow cytometry-based bead array system per manufacturer’s instructions (BioLegend). Paired antibodies were used to conduct sandwich ELISA for the detection of IFN-λ,^39^ IL-6 (BD Biosciences Ab clones MP5-20F3; MP5-32C11), TNF (Peprotech polyclonal Abs 500-p64; 500-P64BT) or ELISA kit for IFN-α (LumiKine, Invivogen) from fresh or frozen tissue culture supernatants.

#### Immunoblotting

For the preparation of soluble cell extracts, harvested cells were lysed in Radioimmunoassay precipitation assay (RIPA) buffer (20 mM Tris, pH 7.4, 150 mM NaCl, 1 mM EDTA, 1% Triton X-100, 0.1% SDS, 10% glycerol, 0.5% sodium deoxycholate, 5 mM NaF, 1 mM Na_3_VO4, 1 mM PMSF, 10 mM NaPPi) then Laemmli buffer (1x concentration, ThermoFisher Scientific), containing benzonase (10 μL/mL) and reducing agent dithiothreitol (0.1 M DDT; ThermoFisher Scientific) was added to the cell extract for 30 min on ice. Cell lysates were then heated at 95°C for 10 min and centrifuged for 15 min at 13,000 rpm. Samples were loaded onto 4–15% TGX Stain free precast protein gels (Bio-Rad Laboratories Pty., Ltd.; California, USA), alongside Precision Plus Dual Xtra Protein Standards (Bio-Rad Laboratories Pty., Ltd.) as molecular weight markers.

Proteins were transferred from SDS-PAGE gels to PVDF membranes with tris-CAPS transfer buffer (Bio-Rad Laboratories Pty., Ltd.) in a sandwich blot by using the trans blot turbo system (Bio-Rad Laboratories Pty., Ltd.). Membranes were probed with primary antibodies at 1:1000, rabbit anti-phospho-STING (ser366) (clone D73CS, Cell Signaling Technology), rabbit anti-STING (clone D2P2F, Cell Signaling Technology), rabbit anti-IRF3 (clone D83B9, Cell Signaling Technology), rabbit-anti phospho irf3 (E6F7Q, Cell Signaling Technology) and anti-Β−actin mab (clone c4, Santa Cruz Biotechnology) in western buffer (TBS with 5% [w/v] skim milk; and 0.05% [w/v] tween 20) overnight at 4°C. Membranes were detected with 1:2000 secondary HRP- conjugated goat anti-rabbit IgG (Cell Signaling Technology), developed with Supersignal Pico (Thermofisher Scientific) per manufactures instructions and imaged using a Chemidoc Touch Imaging System (Bio-Rad Laboratories).

#### Quantitation of bacterial viability

Bacterial viability was quantified using the LIVE/DEAD BacLight Bacterial Viability Kit (Invitrogen, ThermoFisher Scientific) according to an adapted manufacturer’s protocol. Bacteria were incubated with indicated concentrations of lysostaphin in CM for 1h at 37 ͦC. After incubation, samples of the bacterial culture were pelleted in a microcentrifuge tube and resuspended in 0.85% NaCl. Then, a 1:1 mixture of the nucleic acid stains (Syto9 and PI) was added to the sample tubes for 15 min at room temperature, protected from light. Stained bacterial viability was determined in a flow cytometer equipped with a laser emitting at 488 nm.

#### ELISA for detection of cyclic dinucleotides

Bacterial culture supernatants and lysed cell samples were analyzed using the DetectX 3′,3′-Cyclic GAMP (cGAMP) Immunoassay Kit (Arbor Assay, Bioscientific Pty Ltd) and 2′3′-cGAMP Immunoassay Kit (Cayman Chemical, Sapphire Bioscience) according to the manufacturers’ protocols. Optical density (450 nm) was determined using a microtiter plate reader (VersaMax). 2′3′-cGAMP and 3′3′-cGAMP levels were interpolated from the standard curve using a 4-parameter logistic regression generated using Prism (GraphPad Software, La Jolla, USA) as per manufacturer’s protocol.

#### Lentiviral transduction

Lentiviral transduction of MutuDC was performed similarly to a published viral transduction protocol.^62^ Third generation lentivirus was generated by transient transfection of Human Embryonic Kidney 293T (HEK293T) cells with a lentiviral plasmid, pMDLg/pRRE (packaging), RSV-REV (packaging) and VSVg (envelope) plasmids complexed into liposomes using Lipofectamine 2000 (Thermo Scientific) and diluted in OptimMEM (Thermo Scientific). Lentiviruses were harvested 48 h later, filtered through 0.45 μm filters and used to infect MutuDC. Following recovery, MutuDC were subsequently enriched for lentiviral plasmid expression via antibiotic selection or cell sorting.

#### Generation of STING knockout MuTuDC

STING^KO^ MutuDC were generated utilising CRISPR-Cas9 gene editing as described.^63^^,^^64^ Briefly, third generation lentiviral transduction (described above) was used to generate MutuDC expressing Cas9 (Lenti-Cas9-P2A-Blast, Addgene plasmid#73310), which were subsequently enriched by antibiotic selection with blasticidin (InvivoGen). An sgRNA sequence to target murine *Sting* was selected using CRISPick (https://portals.broadinstitute.org/gppx/crispick/public). The sgRNA target sequence (CAGTTGGATGTTTGGCCTTC) was then inserted into the sgRNA backbone, pXPR_BRD003 (Broad Institute). Correct insertion was validated by Sanger sequencing (Monash Micromon). The sgRNA plasmid targeting murine *Sting* was introduced into Cas9 expressing MutuDC via third generation lentiviral transduction before antibiotic selection with puromycin (InvivoGen). Gene disruption was confirmed by immunoblot analysis of STING.

### Quantification and statistical analysis

GraphPad Prism was utilized to graph data and carry out statistical tests, where all test and *p* value details are located in Figure legends. In general, data are typically presented as the mean ± SEM, where a *p* value <0.05 was considered significant as determined by *t* test or Anova as indicated within the specific figure legends. Significance is indicated by asterisks, defined as ∗*p* < 0.05, ∗∗*p* < 0.05 and ∗∗∗*p* < 0.005.
