# Supplementary material for: cGAS/STING sensing in dendritic cells discriminates between daptomycin sensitive and resistant Staphylococcus aureus clinical isolates
Source: iScience. 2026 Apr 22;29(6):115854. doi: 10.1016/j.isci.2026.115854 (PMC13186032; doi:10.1016/j.isci.2026.115854)
Supplement: Document S1. Figures S1–S4, Data S1 and S2 [file mmc1.pdf]

iScience, Volume *29*

Supplemental information

cGAS/STING sensing in dendritic cells discriminates between daptomycin sensitive and resistant *Staphylococcus aureus* clinical isolates

Timothy Patton, Nazneen Jahan, Jhih-Hang Jiang, Xenia Kostoulias, Ee Shan Pang, Rachel J. Lundie, Katherine Balka, Rajan Venkatraman, Viola Oorschot, Joan Clark, Sharifeh Askary, Peck Tan, Angus Shoppee, Georg Ramm, Anton Y. Peleg, Dominic De Nardo, and Meredith O'Keeffe

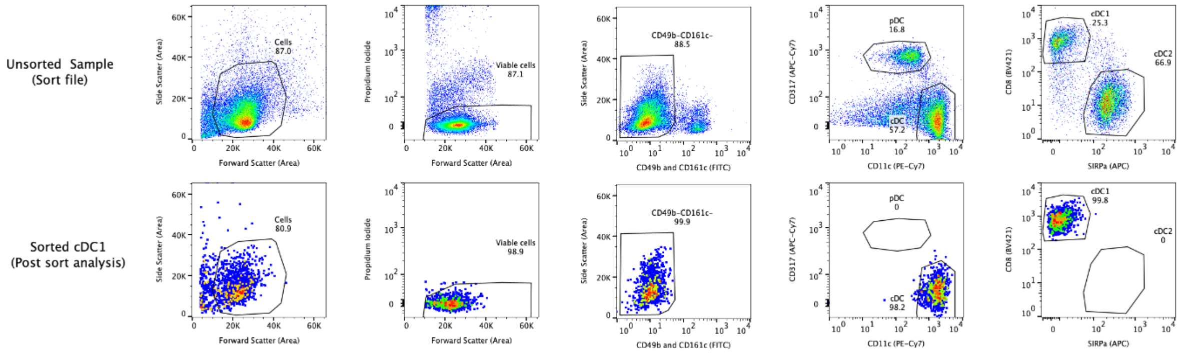

Supplementary Figure 1 Gating strategy for FACS sorting splenic DC subsets. Representative gating strategy used to FACS sort splenic cDC1, cDC2 and pDC for experiments stimulating purified DC subsets with MRSA (as indicated in figure legends). Unsorted spleen cells are shown from sort acquisition (*top*) and post-sort re-analysis of cDC1 are shown (*bottom*).

Fig. S2

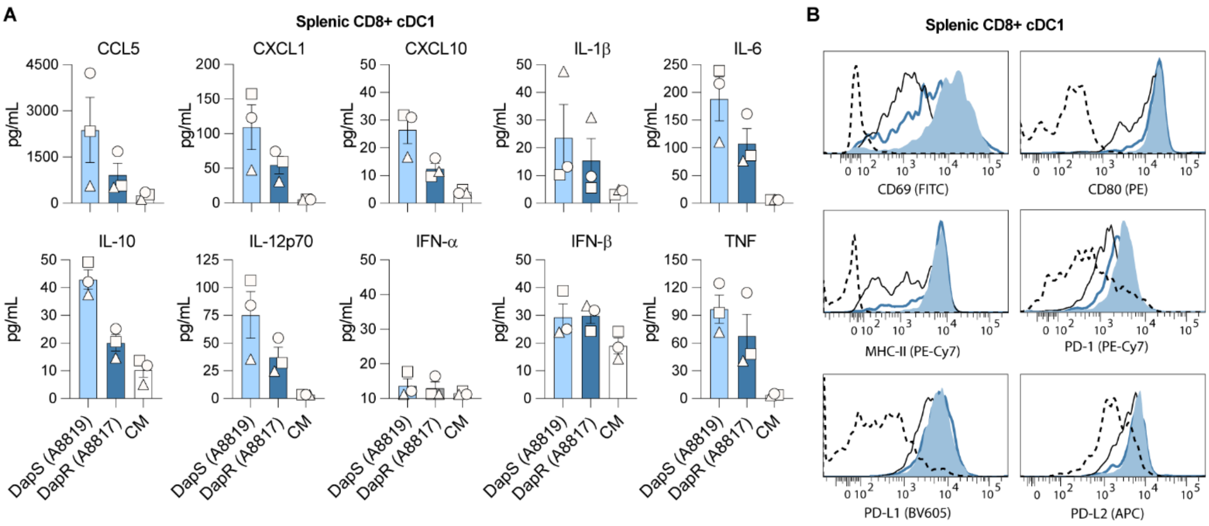

Supplementary Figure 2. Splenic cDC1 differentially recognise paired MRSA clinical isolates

(A) Cytokine secretion (pg/mL) by FACS sorted splenic cDC1 at 18 hours post stimulus with DapS (A8819; light blue), DapR (A8817; dark blue) MRSA (MOI of 10), or media alone control (black). Results from 3 independent experiments are expressed as the mean ± SEM, with each symbol (circle, square and triangle) representing paired experimental replicates (n = 3).

(B) Expression of activation and inhibitory type markers by cDC1 following 18-hour stimulation with DapS (A8819; light blue trace), DapR (A8817; dark blue shaded) MRSA (MOI of 10), or media alone (black solid trace). Pooled sample fluorescence minus one (FMO) control is shown for each marker (black dashed trace). Data shown from one experiment representative of seven (CD80), six (MHC-II), five (PD-1) three (CD69 and PD-L1), and two (PD-L2) independent experiments (n = 2 - 7).

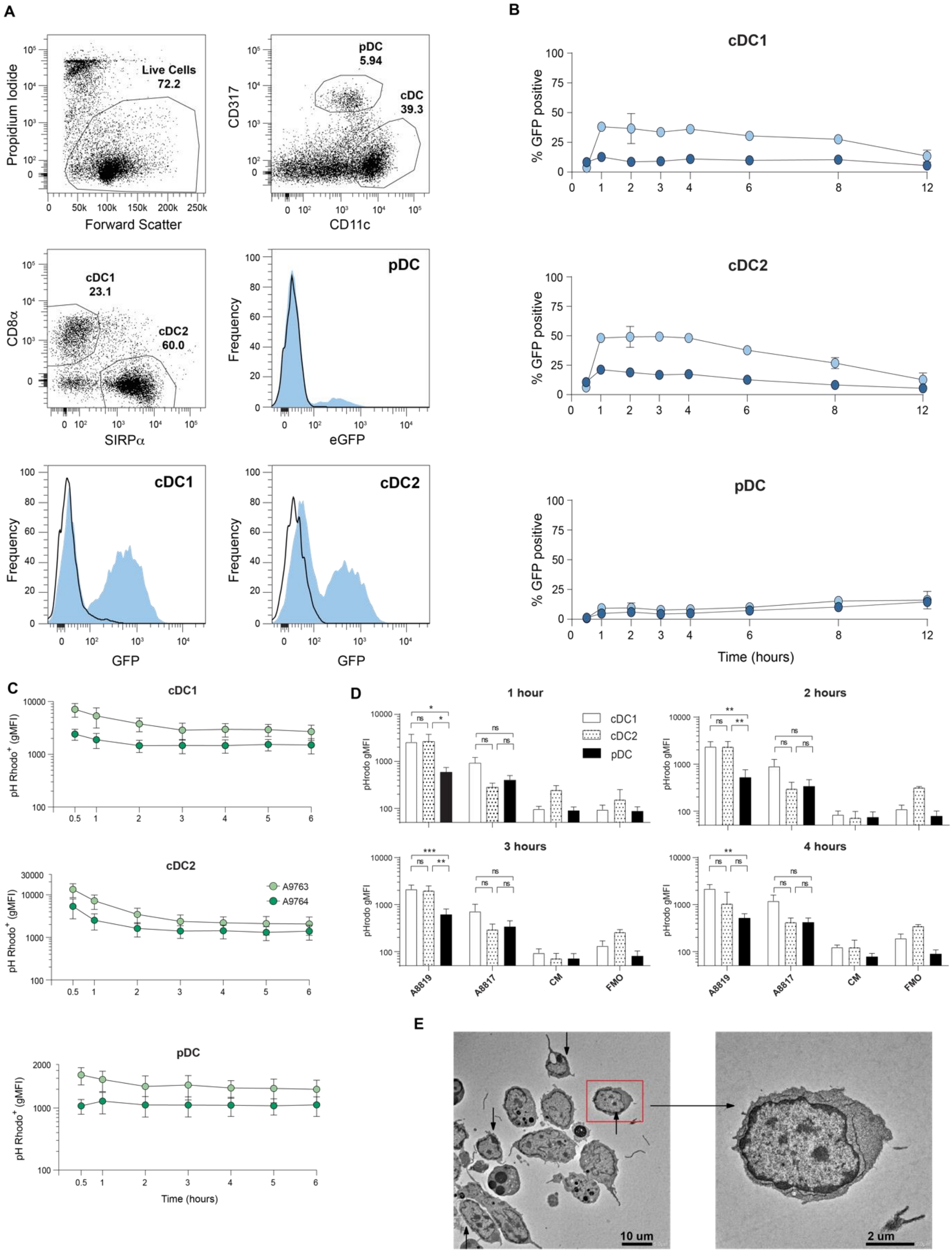

Supplementary Figure 3. Optimisation of *S. aureus* internalisation assays in primary splenic DC

(A) Gating strategy for pre-stained splenic cDC and pDC subsets in bulk cultures with eGFP expressing MRSA strains derived from the DapS (A8819; blue shaded) clinical isolate DapS mock media only control (black trace). Data shown from 1 experiment, representative of 4 independent experiments

(n = 4).

(B) Time course showing percentage of DC subsets positive for eGFP (as in A), as a surrogate marker for internalisation of eGFP expressing bacterial strains. Data points show the mean and range of biological replicates (n = 2).

(C) Time course showing pHrodo gMFI of pre-stained cDC and pDC subsets (as in A) in bulk cultures with pHrodo labelled MRSA clinical isolates DapS (A9763) and DapR (A9764). Data shows the mean and SEM of three independent experiments (n = 3).

(D) pHrodo™ gMFI for cDC1, cDC2 and pDC stimulated over four hours with DapS (A8819), DapR (A8817) MRSA. Significance in panels b and c reflect the results of an ordinary two-way ANOVA, using Tukey's test to correct for multiple comparisons; whereby the degree of significance is defined by $p \leq 0.05$ (*), $p \leq 0.01$ (**) and $p \leq 0.001$ (***); whilst non-significance (ns) is defined by $p > 0.05$. Data show the mean and SEM of biological replicates pooled from four independent experiments (n = 4).

(E) Transmission electron micrograph of fixed and Epon embedded DC sections corresponding to FACS sorted cDC1 following 8-hour MRSA stimuli with DapS A8819 at an MOI of 10. Images were acquired on a JEOL TEM electron microscope with between 200 and 30,000 X magnification. Images show a field of view representative of the sample (left), containing ten live DC entirely within the field of view, of which four do not contain any visible bacteria (indicated by the black arrows), and an increased magnificent of a single DC (right) which does not contain visible MRSA. Scale bars are representative of 2-10 µm as indicated. Data shown from 1 experiment representative of two independent experiments (n = 2).

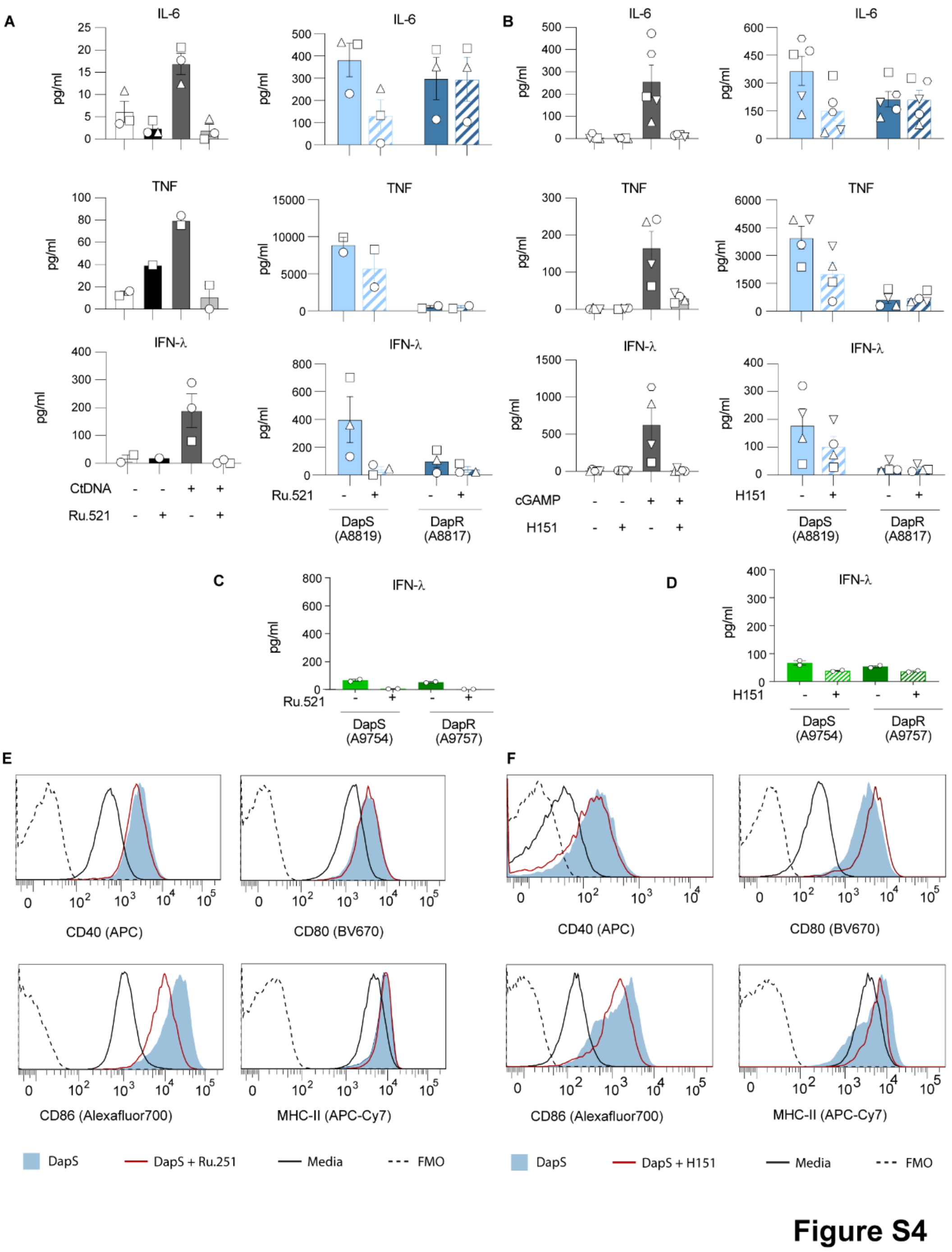

Figure S4. cGAS and STING inhibitors decrease the recognition of DapS *S. aureus* clinical isolates by MutuDC

(A) IL-6, TNF-α and IFN-λ production (pg/mL) by MutuDC, following pre-treatment with the cGAS inhibitor Ru.251 (5μg/mL) or media alone, and then overnight stimulation with ctDNA (5 μg/mL, left graphs) or DapS (A8819) MRSA, DapR (A8817) MRSA (right graphs). Data shows the mean and SEM

pooled from two to three independent experiments (n = 2-3).

(B) IL-6, TNF-α and IFN-λ production (pg/mL) by MutuDC following pre-treatment with 500ng/ml of STING inhibitor H151, and then overnight stimulation with cGAMP as the positive control (left graphs) and DapS (8819) and DapR (8817) MRSA (right graphs). Data shows the mean and SEM pooled from four-five independent experiments (n = 4-5).

(C) IFN-λ production (pg/mL) by MutuDC following pre-treatment with the cGAS inhibitor Ru.251 (5μg/mL) or media alone and then overnight stimulation with alternate MRSA paired clinical isolates, A9754 (DapS) and A9757 (DapR).

(D) IFN-λ production (pg/mL) by MutuDC following pre-treatment with the STING inhibitor H151 (500ng/mL) or media alone, and then overnight stimulation with alternate MRSA paired clinical isolates, A9754 (DapS) and A9757 (DapR).

(E) Expression of CD40, CD80, CD86 and MHC-II by MutuDC following overnight stimulation with DapS MRSA (A8819; light blue shaded), DapS + 5μg/mL Ru.521 (red trace), media alone (black trace) or unstained control (dashed black trace). Data shown from 1 experiment, representative of three independent experiments (n = 3).

(F) Expression of CD40, CD80, CD86 and MHC-II by MutuDC following overnight stimulation with DapS MRSA (A8819; light blue shaded), DapS + 500 ng/mL H151 (red trace), media alone (black trace) or unstained control (dashed black trace). Data shown from 1 experiment, representative of three independent experiments (n = 3).

Data S1: The following data represents original western blot images from Figs 5 and 7, annotated for clarity

Uncropped blot, related to Figure 5 (Original blot)

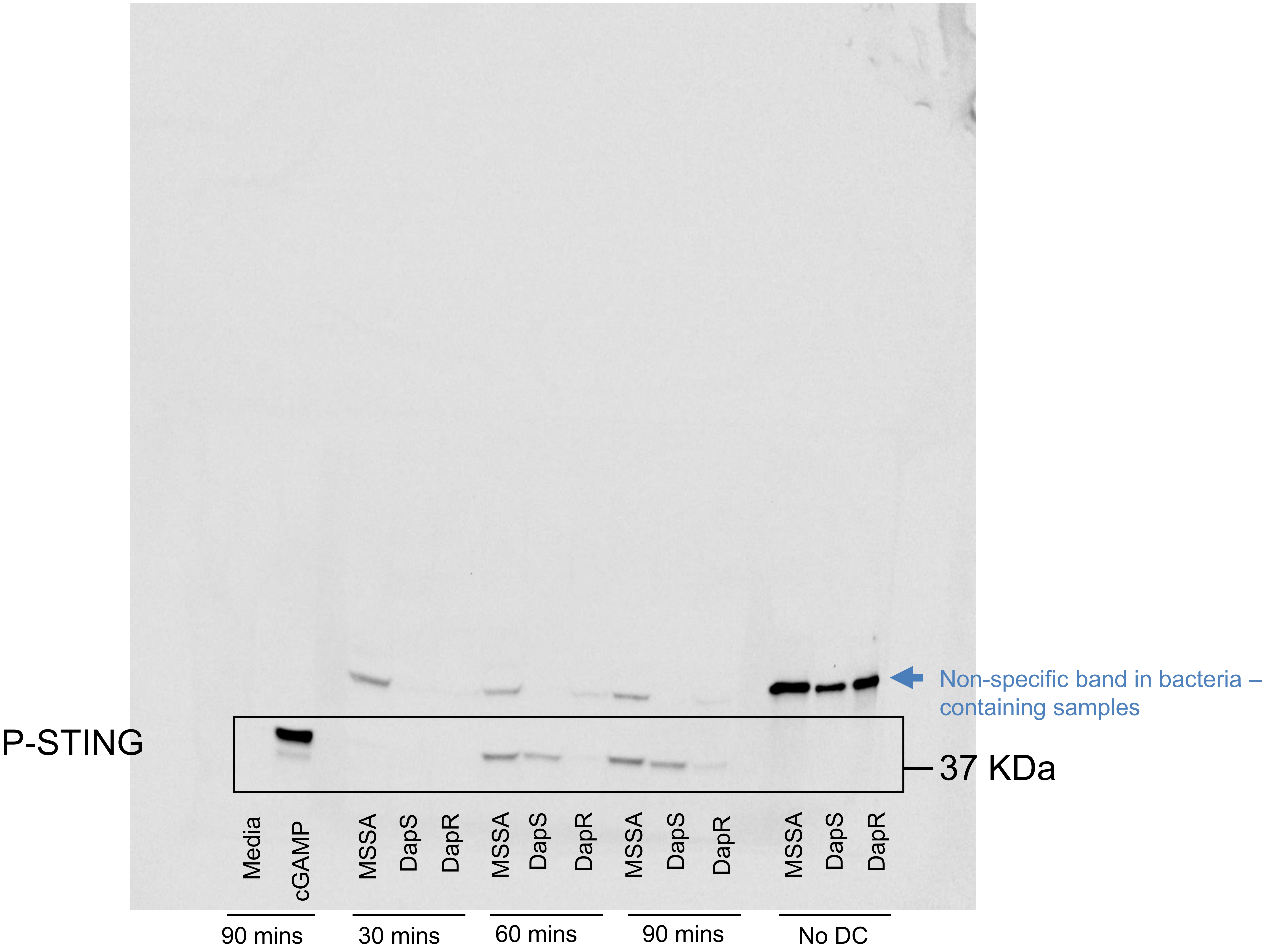

Uncropped blot, related to Figure 7 (Original blots)

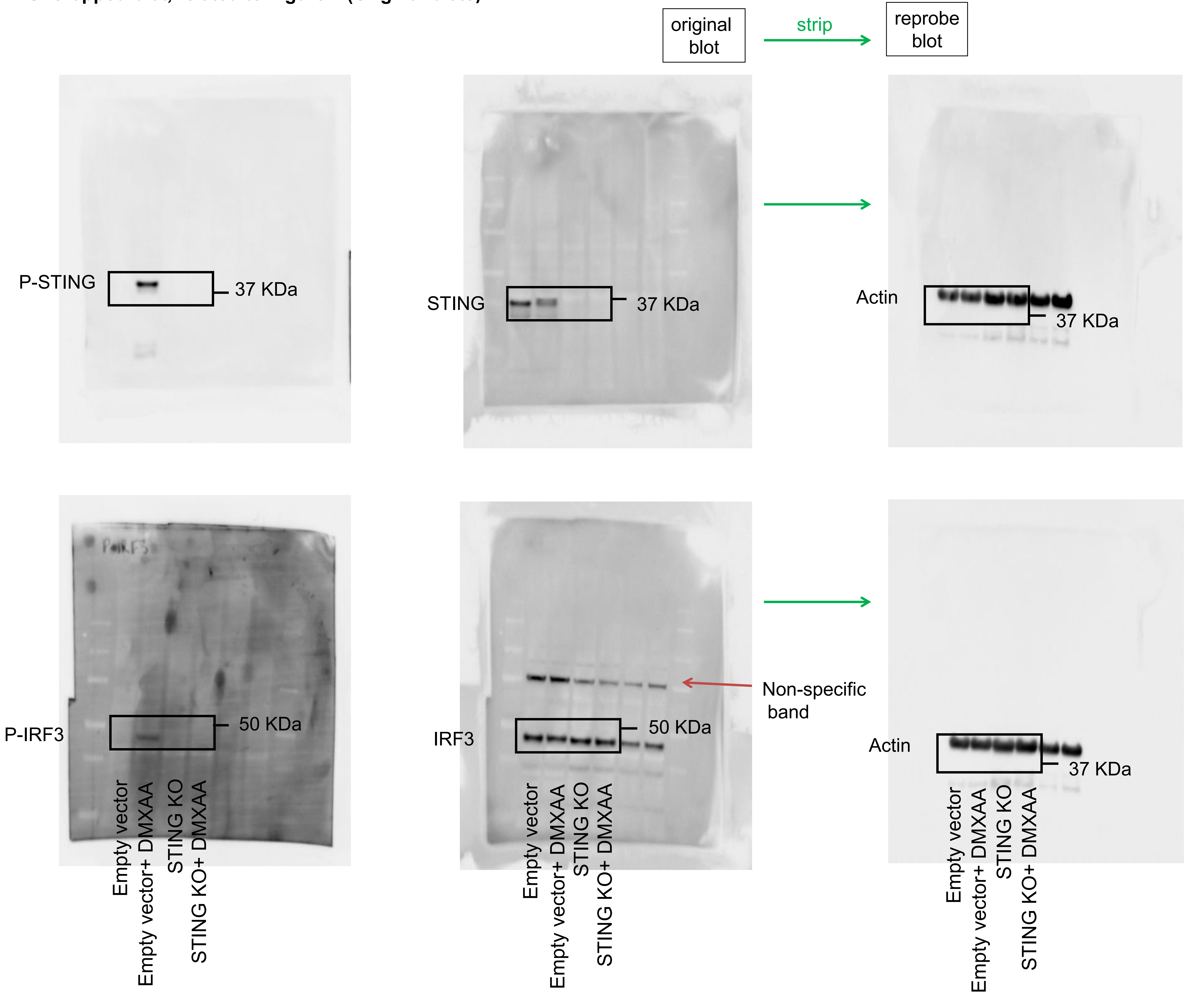

Data S2: JPEG images of Fig 2F DapS and Fig S3E

Fig 2F

DapS

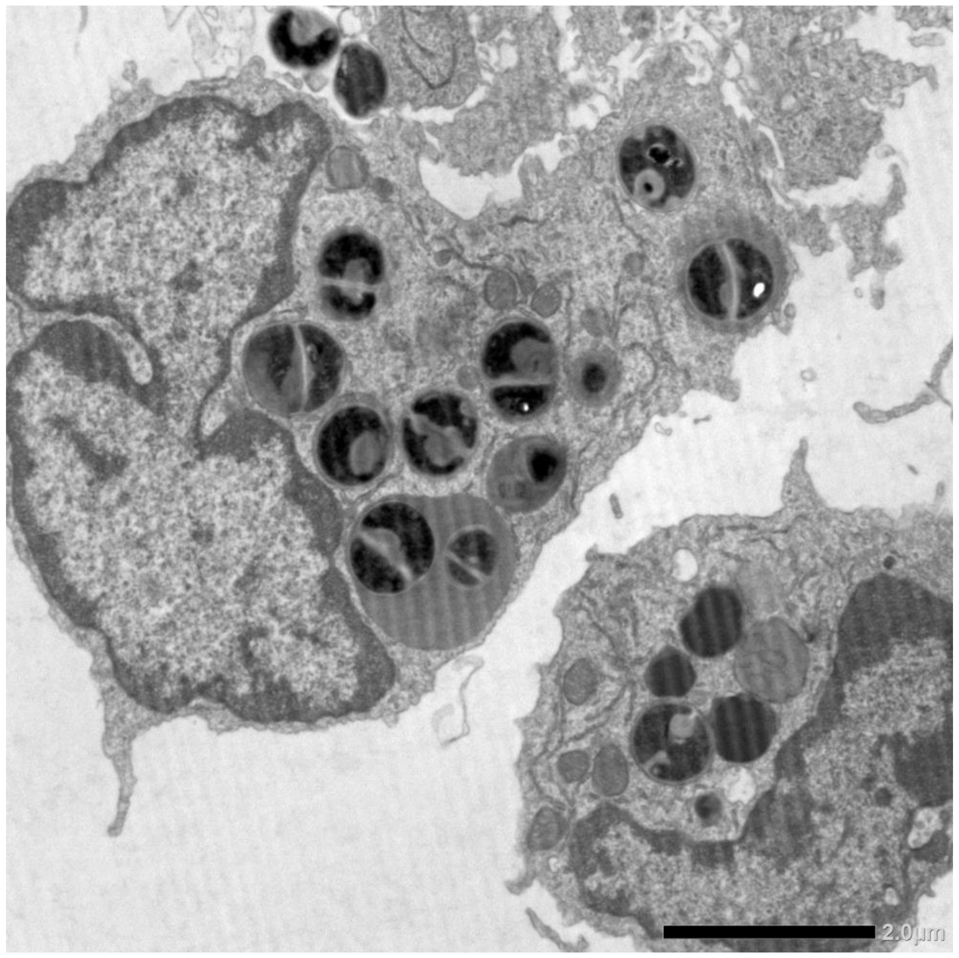

Grid N1 Section A_029_SA-MAG_X4000

DapR

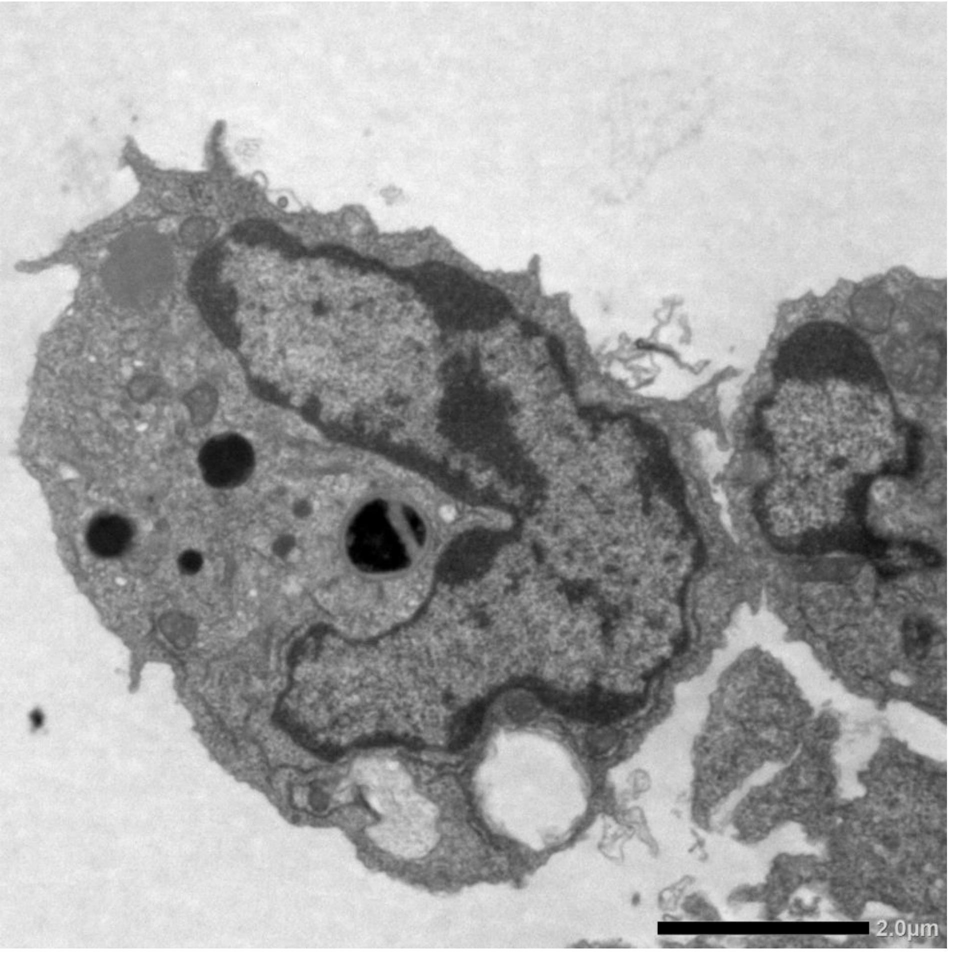

Grid Q2 Section G_010_SA-MAG_X4000

Fig S3E

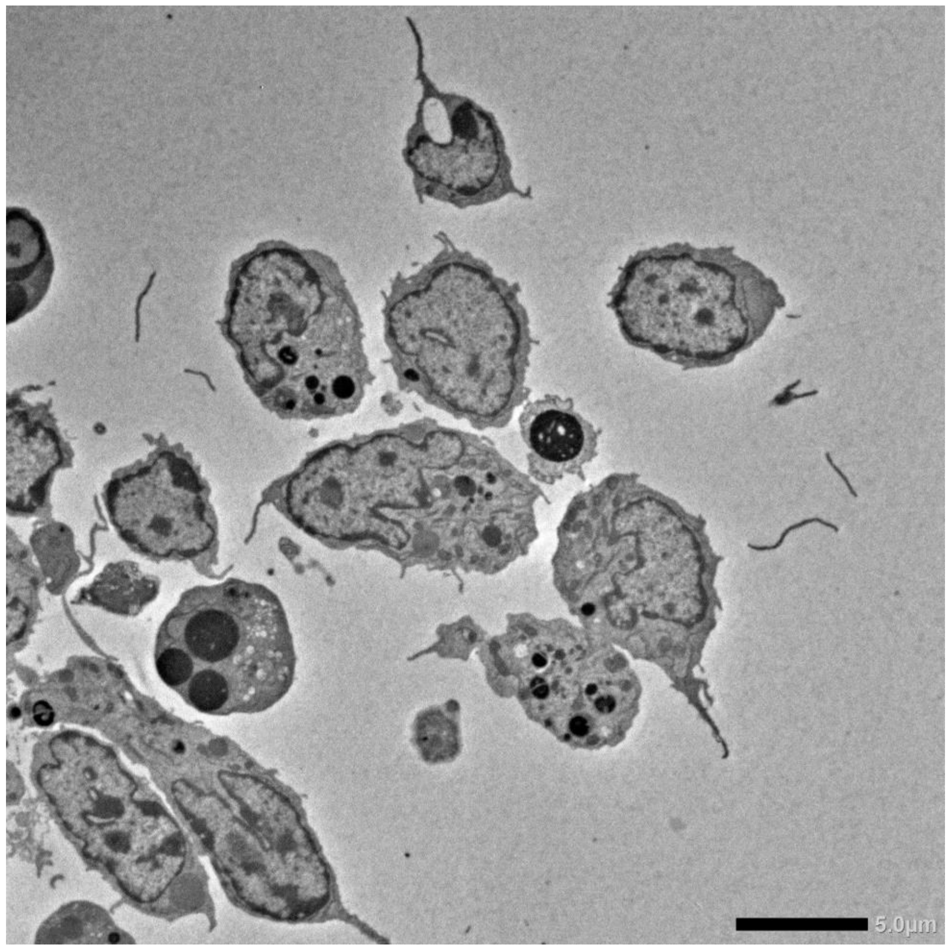

M2 A A8819_MAG_X1000_034

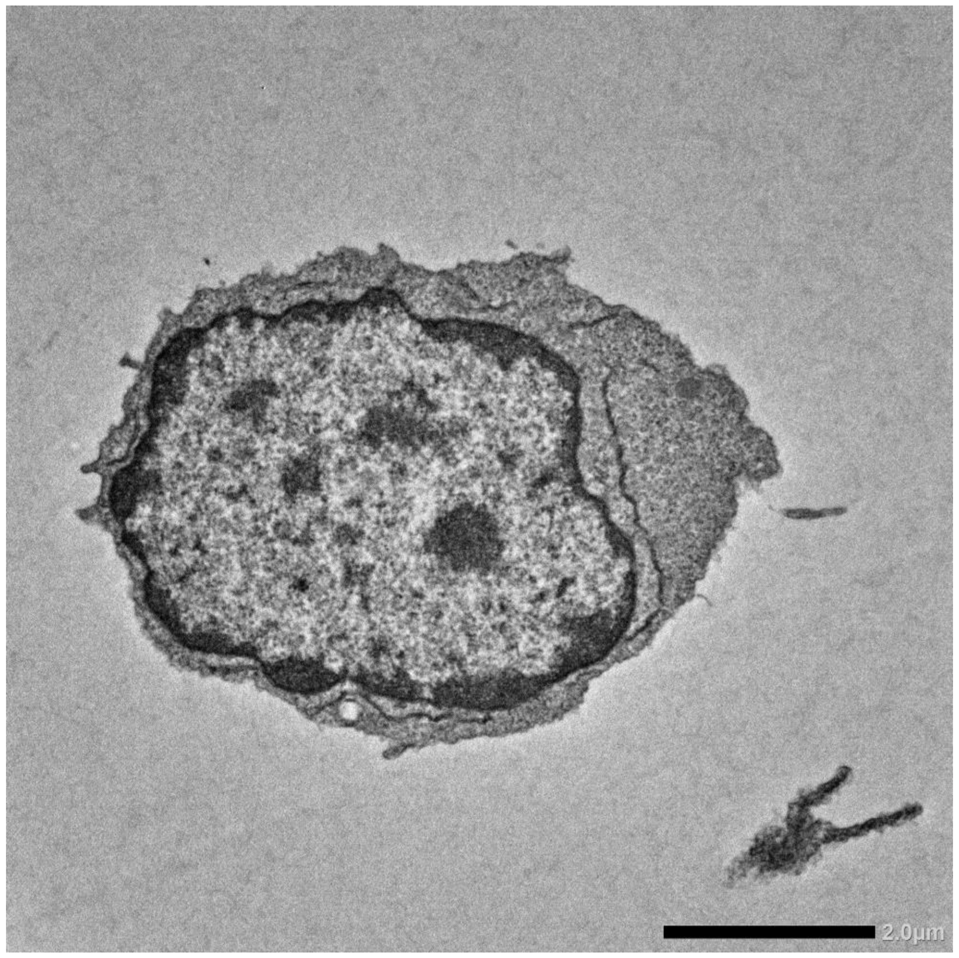

M2 A A8819_SA-MAG_X4000_048
